# Suppression of Skp2 contributes to sepsis-induced acute lung injury by enhancing ferroptosis through the ubiquitination of SLC3A2

**DOI:** 10.1007/s00018-024-05348-3

**Published:** 2024-07-30

**Authors:** Zhaoyuan Chen, Jie Zhang, Shenjia Gao, Yi Jiang, Mengdi Qu, Jiahui Gu, Han Wu, Ke Nan, Hao Zhang, Jun Wang, Wankun Chen, Changhong Miao

**Affiliations:** 1 Department of Anesthesiology, Zhongshan Hospital, Fudan University, 180# Feng-Lin Road, Shanghai, 200032 China; 2 Shanghai Key Laboratory of Perioperative Stress and Protection, Shanghai, China; 3 Department of Integrative Medicine and Neurobiology, School of Basic Medical Science, Shanghai Medical College, Fudan University, Shanghai, 200032 China; 4 Department of Anesthesiology, QingPu Branch of Zhongshan Hospital Affiliated to Fudan University, 1158# Gongyuan Dong Road, Shanghai, 201700 China

**Keywords:** Sepsis, Skp2, Ferroptosis, Cytokine storm, SLC3A2

## Abstract

Sepsis is a life-threatening organ dysfunction caused by a dysregulated host response to infection. The inflammatory cytokine storm causes systemic organ damage, especially acute lung injury in sepsis. In this study, we found that the expression of S-phase kinase-associated protein 2 (Skp2) was significantly decreased in sepsis-induced acute lung injury (ALI). Sepsis activated the MEK/ERK pathway and inhibited Skp2 expression in the pulmonary epithelium, resulting in a reduction of K48 ubiquitination of solute carrier family 3 member 2 (SLC3A2), thereby impairing its membrane localization and cystine/glutamate exchange function. Consequently, the dysregulated intracellular redox reactions induced ferroptosis in pulmonary epithelial cells, leading to lung injury. Finally, we demonstrated that intravenous administration of Skp2 mRNA-encapsulating lipid nanoparticles (LNPs) inhibited ferroptosis in the pulmonary epithelium and alleviated lung injury in septic mice. Taken together, these data provide an innovative understanding of the underlying mechanisms of sepsis-induced ALI and a promising therapeutic strategy for sepsis.

**Supplementary Information:**

The online version contains supplementary material available at 10.1007/s00018-024-05348-3.

## Introduction

Sepsis is a life-threatening disease related to a dysregulated host immune response to infection. The activation of innate immune cells including neutrophils and macrophages, plays a crucial role in the excessive secretion of cytokines and hence the development of cytokine storms during sepsis [1]. Cytokine storms trigger the onset of multiorgan damage or dysfunction syndrome, which is the major cause of sepsis-related death. Sepsis-induced acute lung injury (ALI) is the most common complication and has a high mortality rate if not treated in a timely manner [2]. Inflammatory cytokine storms are known to induce programmed cell death and interfere with gas exchange in lung epithelial cells [3], leading to lung injury. However, the essential regulatory components that are responsible for inducing cell death in the alveolar epithelium and the regulatory mechanisms involved in sepsis-induced ALI remain to be determined.

Ferroptosis is an iron-dependent form of programmed cell death caused by the accumulation of lipid peroxides [4], and is induced when the oxidative load in a cell surpasses its internal antioxidant system. The canonical ferroptosis axis is initiated by blocking the import of cystine via the cystine-glutamate antiporter system Xc^−^ (composed of SLC7A11 and SLC3A2), followed by decreased biosynthesis of reduced glutathione (GSH). A decrease in the levels of GSH, a powerful reductant and a cofactor for glutathione peroxidase 4 (GPX4), impairs GPX4-mediated regulation of lipid homeostasis, thereby disrupting the cellular redox balance and consequently ferroptosis [5]. Ferroptosis actively participates in many pathological conditions, such as tumors, neurological diseases, acute kidney injury, and ischemia/reperfusion [6]. Our previous study revealed the occurrence of ferroptosis in alveolar epithelial cells during sepsis and revealed its contribution to the pathogenesis of sepsis-induced ALI [7], suggesting that targeting ferroptosis could be a potential strategy for the treatment of sepsis-induced ALI. However, the underlying mechanisms regulating ferroptosis during sepsis-induced ALI remain unclear.

Varying levels of posttranslational modifications, including ferroptosis, are key to the dynamic regulation of programmed cell death. Ubiquitination is a dynamic and reversible posttranslational modification that could be a promising therapeutic target for treating inflammatory diseases including lung injury [8]. It plays an active role in the activation of pro- and anti-inflammatory signaling pathways [1] and modulates the initiation and implementation of cell death pathways [9]. A previous study revealed that the E3 ubiquitin ligase MARCHF6 mediates the degradation of key ferroptosis effectors and therefore downregulates ferroptosis, indicating that ubiquitination is intricately linked to the regulation of ferroptosis [10]. S-phase kinase-associated protein 2 (Skp2) is an E3 ubiquitin ligase that mediates the targeting of substrates for proteasomal degradation via ubiquitination [11]. Furthermore, Skp2 can interact with polyubiquitin chains and thus regulate protein localization, downstream transcriptional events, and pathway activation through its nonproteolytic function [12]. However, whether Skp2 plays a role in inducing ferroptosis during sepsis-induced ALI remains to be explored.

In the present study, we utilized patient samples and a septic mouse model to investigate the impact of the absence of Skp2 on sepsis-induced acute lung injury (ALI) through the promotion of ferroptosis. Mechanistically, Skp2 promoted the ubiquitination of SLC3A2, an upstream ferroptosis protein, via a K48-linked nondegradative mechanism and thereby regulated the normal transport of cystine and glutamate. In sepsis, inflammatory cytokine storms suppress Skp2 expression, leading to reduced SLC3A2 ubiquitination and disruption of the translocation of cysteine and glutamate. An increased oxidative stress response and ferroptosis are ultimately triggered in lung epithelial cells. To further explore the therapeutic potential of Skp2, lipid nanoparticle (LNP) delivery systems [13] were developed to enable the targeted delivery of Skp2 mRNA to murine lungs. The overexpression of the Skp2 protein in the lungs significantly reduced mortality and ameliorated lung injury in septic mice. These results indicate that overexpressing Skp2 through the LNP system could be a potential therapeutic strategy for sepsis-induced ALI.

## Results

### Cytokine storm during sepsis inhibited Skp2 expression in lung epithelial cells

**Fig. 1:**
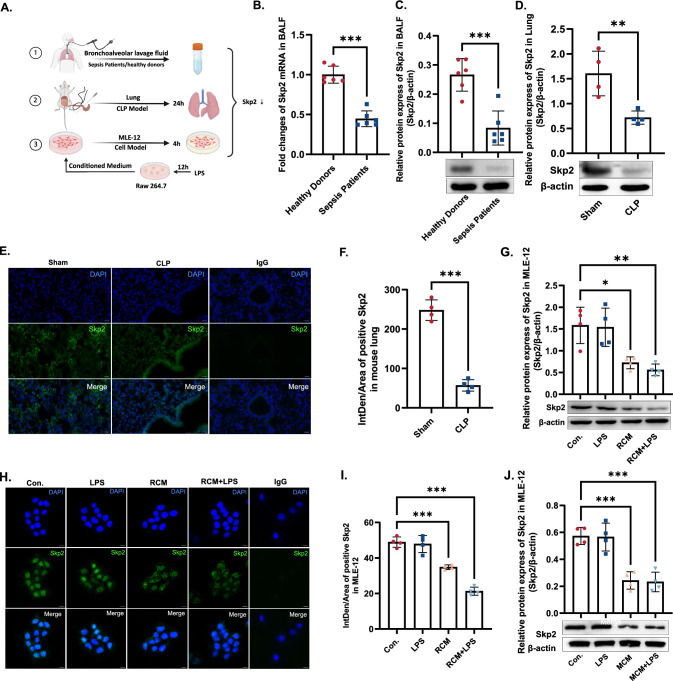
Cytokine storm during sepsis inhibited Skp2 expression in lung epithelial cells. **A** Experimental setup and sample collection (generated by Biorender). BALF samples were collected from septic patients and healthy donors (n = 6). C57BL/6 mice were randomly divided into the sham or CLP sepsis groups (n = 4). The murine monocyte/macrophage line Raw264.7 was stimulated with LPS and the culture medium was collected after 12 h (from Raw264.7 cell-conditioned medium, RCM). MLE-12 murine lung epithelial cells were treated with RCM or LPS for 4 h. **B**, **C** The mRNA and protein expression of Skp2 in the BALF of septic patients and healthy donors were measured by qPCR (**B**) and western blotting (**C**). **D**–**F** Lung tissue of mice was collected 24 h after CLP surgery, and the protein expression of Skp2 in the lungs was measured by western blotting (**D**) or immunofluorescence staining (**E**, **F**). Scale bar = 20 μm. **G**–**I** The following four groups were included in the in vitro experiment: control group (Con.), LPS treatment group (LPS), Raw264.7 conditioned medium treatment group (RCM), and RCM plus LPS costimulation group (RCM + LPS). MLE-12 cells were stimulated for 4 h, after which Skp2 protein expression was measured by western blotting (**G**) and immunofluorescence staining (**H**, **I**). Scale bar = 20 μm. **J** The mouse lung resident macrophage line MH-S was stimulated with LPS and the cell culture supernatant were collected as MH-S-conditioned medium (MCM). MLE-12 cells were then divided into the following groups: control group (Con.), LPS treatment group (LPS), MH-S conditioned medium stimulation group (MCM), and MCM plus LPS costimulation group (MCM + LPS). The data are presented as the means ± standard deviations (*P < 0.05, **P < 0.01, ***P < 0.001)

To determine the expression of Skp2 in sepsis, patient samples and a mice model of sepsis were utilized. The experimental setup and sample collection are shown in Fig. 1A. Briefly, bronchoalveolar lavage fluid (BALF) was collected from septic patients and healthy donors, and C57BL/6 mice were randomly divided into the sham or cecal ligation and puncture (CLP)—induced sepsis groups. First, a notable reduction in the expression of Skp2 mRNA and protein was observed in the BALF of septic patients, compared to that in the BALF of healthy donors (Fig. 1B, C). Epithelial cells accounted for approximately 5% of the cell population in BALF. The number of epithelial cells in BALF tends to increase in response to infection [14]. This observation suggests a plausible link between the Skp2 protein and sepsis-induced ALI. Using a CLP septic mouse model, we also observed a significant reduction in Skp2 expression in the lungs of CLP mice (Fig. 1D–F, Supplemental Fig. 1A), compared to that in the lungs of control mice. As reported, the BALF contains a mass of epithelial cells [15], as does was the lung from a mouse model of human disease, and we wondered whether the reduction in Skp2 was mainly in epithelial cells.

To explore this hypothesis, MLE-12 murine lung epithelial cells were used as a cell model. During the course of lung infection, activated alveolar macrophages secrete large amounts of cytokines which further trigger an intricate inflammatory cascade, resulting in subsequent pulmonary epithelial cell death, which is directly related to sepsis-induced ALI [16]. Thus, lipopolysaccharide (LPS)-activated Raw264.7 cell-conditioned medium (RCM) was used to mimic the in vivo inflammatory conditions of sepsis (Fig. 1A). We first evaluated cytokine levels in RCM and found that after LPS stimulation, macrophages secreted higher levels of IL-6, TNF-α, and MCP-1, while no significant changes were observed in IFN-γ, IL-10, IL-12 or MCP-1 (Supplemental Fig. 1C–I). Since IFN-γ does not serve as the primary factor secreted by macrophages, no detectable alterations were detected. After treatment with RCM, the expression of Skp2 in MLE-12 cells was dramatically reduced, as expected (Fig. 1G–I, Supplemental Fig. 1B). Similar findings were observed in the conditioned medium of the LPS-stimulated murine MH-S alveolar macrophage line (Fig. 1J). Together, these results suggest that inflammatory cytokines may be responsible for the decrease of Skp2 in sepsis. Previous studies have showed that TNF-α [17] or IFN-γ [18] can induce ferroptosis. Although Skp2 downregulation in lung epithelial cells was achieved solely through treatment with TNF-α or IFN-γ (Supplemental Fig. 1J), subsequent experiments involving treatment with RCM were used to better mimic the in vivo impact of the cytokine storm.

### Skp2 deficiency induced ferroptosis of alveolar epithelial cells during sepsis

**Fig. 2:**
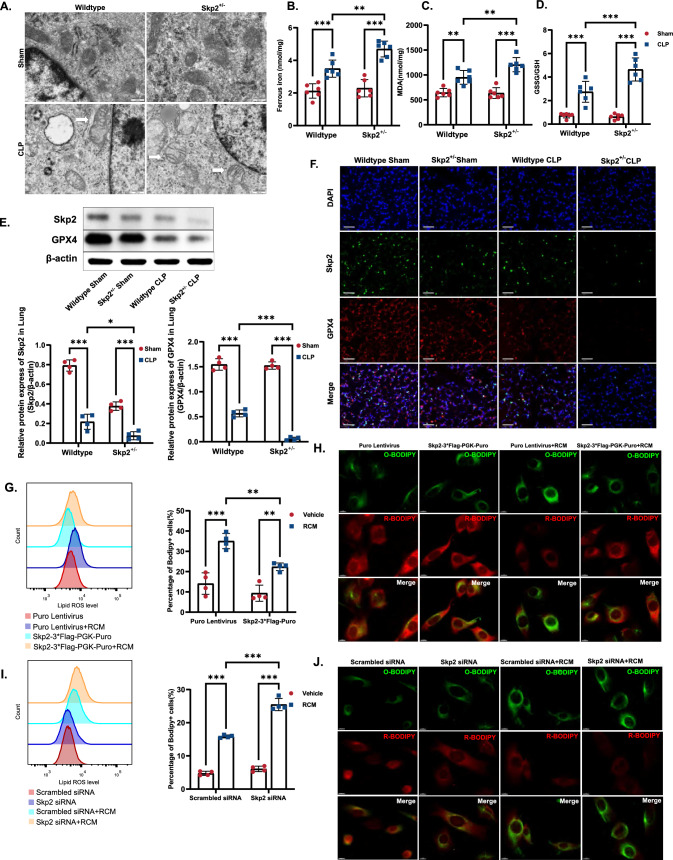
Skp2 deficiency induced ferroptosis of alveolar epithelial cells during sepsis. Wildtype and Skp2^+/−^ C57BL/6 mice were randomly assigned to the sham or CLP group (n = 4 for Western blotting, n = 6 for other analyses). **A** Pulmonary mitochondrial ultrastructure was visualized by electron microscopy, scale bar = 500 μm. **B** The Fe^2+^ levels in 10 mg lung tissues were examined with iron assay kit. **C** Lipid peroxidation was examined by measuring the MDA content in the lungs. **D** The GSSG/GSH ratio in the lung was measured with a GSSG/GSH quantification kit. The relative protein expression of GPX4 and Skp2 in the lungs was measured by western blotting (**E**) and immunofluorescence staining (**F**). **G**, **H** MLE-12 lung epithelial cells were transfected with Skp2 lentivirus (Skp2-3*Flag-PGK-Puro) to overexpress Skp2 or with control lentivirus, and the transfected cells were then selected with puromycin. **G** Lipid peroxidation was measured by flow cytometry using Bodipy. **H** The fluorescent dye Bodipy was used to visualize lipid peroxidation (green) and reduction (red) under a fluorescence microscope, scale bar = 10 μm. **I**, **J** Skp2 in MLE-12 cells was knocked down by siRNA, and the cells were treated with RCM for 4 h. **I** Cells transfected with the lipid peroxidation probe Bodipy were analyzed by flow cytometry for the detection of positive cells. **J** The fluorescent dye Bodipy was used to visualize lipid peroxidation (green) and reduction (red) under a fluorescence microscope. Scale bar = 10 μm. The data are presented as the means ± standard deviations (*P < 0.05, **P < 0.01, ***P < 0.001)

Our previous study [7] provided evidence for ferroptosis in lung epithelial cells during sepsis. Here, we aimed to investigate the impact of Skp2 on pulmonary epithelial ferroptosis and sepsis-induced ALI. Since Skp2^−/−^ mice have lower body weights than their Skp2^+/−^ counterparts[19], often die shortly after birth and exhibit physical abnormalities[20], we used heterozygous Skp2^+/−^ mice in our subsequent experiments. Morphological features of ferroptosis were observed in the lungs of Skp2^+/−^ CLP mice (Fig. 2A) via electron microscopy, as indicated by mitochondrial shrinkage, increased density of mitochondrial membranes, and compromised membrane integrity [21]. At the molecular level, ferroptosis is characterized by the accumulation of intracellular ferrous iron and lipid peroxidation, an increased ratio of GSSG (the oxidative status of GSH) to GSH and decreased expression of GPX4. Therefore, we performed a series of tests to evaluate the redox state and iron accumulation within the lungs. Specifically, we measured ferrous ion concentrations (Fig. 2B), lipid oxidation (MDA quantification) (Fig. 2C), and the GSSG/GSH ratio (Fig. 2D). Our findings revealed a notable increase in these parameters in Skp2^+/−^ CLP septic mice, compared to those in wildtype CLP mice. Moreover, we observed a significant reduction in the expression of GPX4, an essential enzyme of ferroptosis, in the lungs of the Skp2^+/−^ mice, compared to the lungs of the wildtype CLP mice (Fig. 2E, F). These data suggest that the depletion of Skp2 is associated with the increase in ferroptosis in sepsis.

To elucidate the role of Skp2 in facilitating ferroptosis of the lung epithelium, Skp2 was overexpressed or knocked down in MLE-12 cells. The Bodipy probe was utilized as an indicator of lipid peroxide levels. This probe allowed for both qualitative visualization (lipid peroxidation in green and reduction in red) and quantitative assessment via flow cytometry. Consistently, treatment with RCM resulted in the induction of ferroptosis, as evidenced by lipid accumulation (Fig. 2G, H) observed using Bodipy probes, along with the downregulation of GPX4 (Supplemental Fig. 2A). The overexpression of Skp2 conferred resistance to RCM-induced ferroptosis. Conversely, siRNA-mediated Skp2 knockdown promoted the peroxidation of intracellular lipids (Fig. 2I, J) and facilitated ferroptosis in MLE-12 cells (Supplemental Fig. 2C). Similarly, treatment with the Skp2 inhibitor SMIP004 depleted GPX4 (Supplemental Fig. 2D) and induced lipid peroxidation (Supplemental Fig. 2F), thus exacerbating ferroptosis. Taken together, these data indicate that Skp2 deficiency is responsible for inflammatory cytokine-induced ferroptosis in lung epithelial cells during sepsis.

### RCM-induced activation of the MEK-ERK pathway mediated Skp2 inhibition in alveolar epithelial cells

**Fig. 3:**
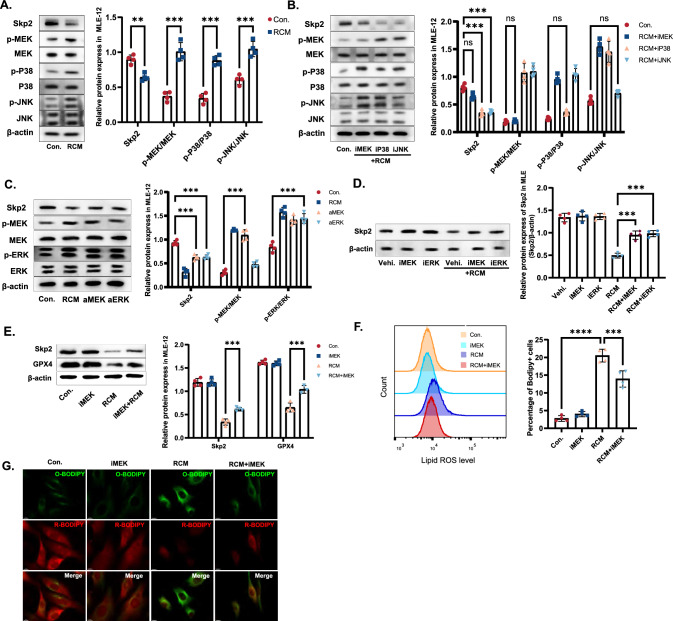
RCM-induced activation of the MEK-ERK pathway mediated Skp2 inhibition in alveolar epithelial cells. **A** MLE-12 cells were treated with RCM, and changes in Skp2 and the MEK/ERK pathway, the p38 pathway, and the JNK pathway were measured by western blotting. **B** MLE-12 cells were subjected to treatment with RCM and specific inhibitors targeting the MEK, p38, and JNK signaling pathways. Alterations in protein expression levels in MLE-12 cells were assessed using western blot analysis. **C** MLE-12 cells were treated with RCM and a specific activator targeting MEK/ERK (C16-PAF and (rel)-AR234960), and the protein levels were measured by western blotting. **D** MLE-12 cells were treated with RCM and/or a MEK/ERK inhibitor (AZD8330/FR180204). The relative protein expression of MEK/ERK kinase pathway and Skp2 was measured by western blotting. **E** Western blotting was used to measure the expression of Skp2 and GPX4 in mouse lung epithelial cells. **F** Cells transfected with the lipid peroxidation probe Bodipy were analyzed by flow cytometry for the detection of positive cells. **G** The fluorescent dye Bodipy was used to visualize lipid peroxidation (green) and reduction (red) under a fluorescence microscope. Scale bar = 10 μm. The data are presented as the means ± standard deviations (ns* P* > 0.05, *P < 0.05, **P < 0.01, ***P < 0.001)

Changes in the internal environment during sepsis are mediated by multiple factors, including the MEK/ERK pathway, p38 pathway, and JNK pathway, which are classical pathways activated by inflammation [22]. Pathogens or pathogen components activate the three pathways to modulate cellular and humoral immunity. These pathways orchestrate a complex cascade of events involving various mediators that profoundly impact humoral immunity. Stimulation of alveolar epithelial cells with RCM confirmed the activation of three pathways in an inflammatory environment (Fig. 3A). Repression of only the MEK pathway, however, affected on Skp2 expression (Fig. 3B). In previous studies, the MEK-ERK kinase pathway was shown to exert bidirectional regulatory effects on the expression, activity, and stability of E3 ubiquitin ligases [23, 24], as well as deubiquitinating enzymes [25]. Activation of ERK alone is sufficient to the induce downregulation of Skp2 [26, 27].

Our validation revealed that the activation of MEK/ERK phosphorylation by inflammatory factors leads to the downregulation of Skp2 (Fig. 3A). To establish a comprehensive understanding of this relationship, we modulated the MEK-ERK kinase pathway using various inhibitors and agonists, resulting in distinct outcomes in terms of ferroptosis (Fig. 3C). MEK/ERK inhibitors [28, 29] have the potential to rescue Skp2 inhibition and attenuate ferroptosis caused by inflammation (Fig. 3D). Based on the aforementioned findings, our observations suggest that inflammation leads to the inhibition of Skp2 by activating the MEK-ERK pathway, and that the absence of Skp2 intensifies ferroptosis, consequently exacerbating sepsis-induced ALI (Fig. 3E–G). This study revealed the existence of a regulatory cascade in inflammation and Skp2.

### Skp2 deficiency aggravated sepsis-induced ALI via the induction of ferroptosis

**Fig. 4:**
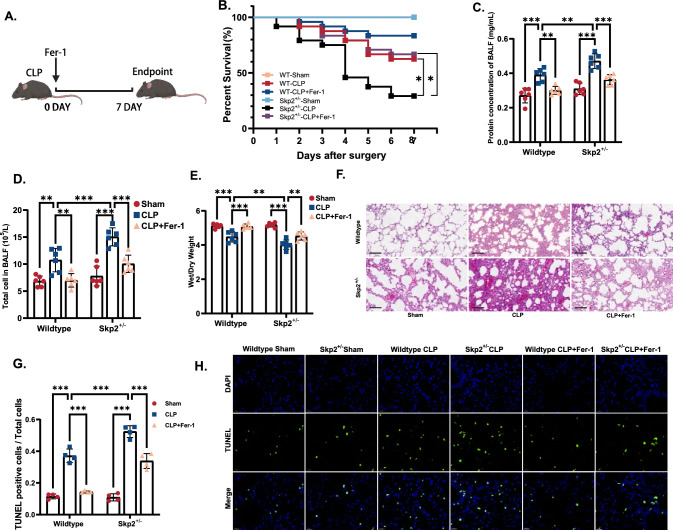
Skp2 deficiency aggravated sepsis-induced ALI via the induction of ferroptosis. **A** Experimental setup for C57BL/6 (generated by Biorender). Wildtype and Skp2^+/−^ C57BL/6 mice were randomly divided into the sham, CLP and CLP + Fer-1 groups, C57BL/6 mice were intravenously injected with Fer-1 (3 mg/kg). n = 24 for survival analysis, n = 6 for other analyses. Survival curve analysis was conducted to evaluate the 7 day survival rate of the mice, while the remaining measurements were carried out at the 24-h time point. **B** Kaplan–Meier survival analysis of the mice. **C** The protein concentration in BALF (mg/mL) was determined by BCA Protein Assay Kits. **D** Total cell numbers in BALF were counted. **E** Wet/dry weight ratio of lung tissues. **F** H&E staining of lung tissues, scale bar = 100 μm. **G**, **H** TUNEL staining of lung tissues (scale bar = 20 μm) and quantification of TUNEL-positive cells among the total cells in each group. The data are presented as the means ± standard deviations (*P < 0.05, **P < 0.01, ***P < 0.001)

Compared with the wild-type CLP mice, the Skp2^+/−^ CLP-induced septic mice exhibited a considerably greater mortality rate (Fig. 4A, B). We then further evaluated the degree of lung injury induced by sepsis. Lung injury is characterized by increased permeability of the alveolocapillary membrane which results in the release of immune cells and proteins into BALF, subsequently leading to edema and diffuse alveolar damage [30]. Therefore, we typically assess the protein concentration and number of nucleated cells in BALF, the lung wet/dry ratio, and the extent of alveolar damage [31]. As shown in Fig. 4C–E, the absence of Skp2 led to a significant increase in the protein concentration and total cell number in BALF, as well as a decrease in the wet/dry ratio, indicating that Skp2 deficiency exacerbated sepsis-induced ALI. HE staining (Fig. 4F) and TUNEL staining [32] (Fig. 4G, H) further revealed aggravated pulmonary injury and elevated chromosomal DNA fragmentation in the lungs of the Skp2^+/−^ CLP-induced septic mice, compared to those of the wildtype CLP mice. However, sepsis-induced ALI aggravated by Skp2 deficiency was significantly attenuated by the administration of ferroptosis inhibitor Ferrostatin-1 (Fer-1).

### Skp2 promoted the ubiquitination of SLC3A2 via a K48-linked nondegradative mechanism

**Fig. 5:**
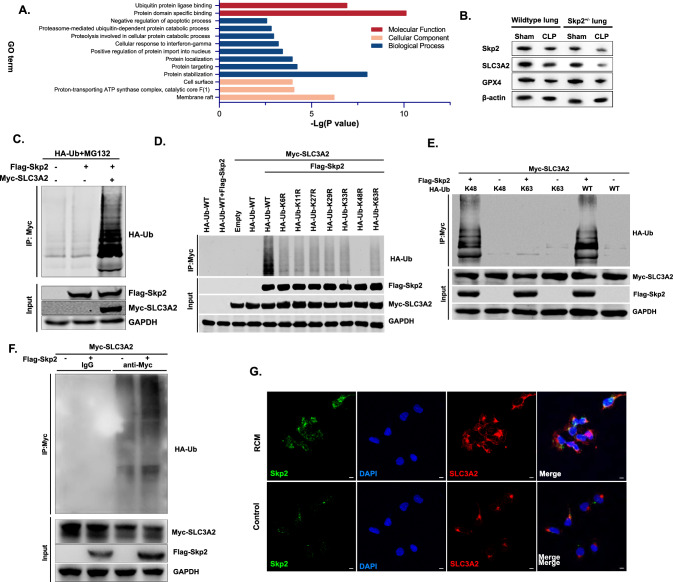
Skp2 promoted the ubiquitination of SLC3A2 via a K48-linked nondegradative mechanism. **A** MLE-12 cells overexpressing Skp2 were subjected to immunoprecipitation-mass spectrometry (IP-MS). The identified proteins were annotated with GO terms. **B** The expression levels of Skp2, GPX4 and SLC3A2 in the lungs of wildtype/Skp2^+/−^ Sham and CLP mice were measured by western blotting. **C** HEK293T cells were transfected with Flag-Skp2, Myc-SLC3A2, or HA-Ub and treated with MG132, and the IP samples and whole cell lysates were analyzed by immunoblotting. **D** HEK293T cells were transfected with different mutants, and the IP samples and whole cell lysates were analyzed by immunoblotting. **E** HEK293T cells were transfected with Flag-Skp2, Myc-SLC3A2, or K48/K63/wildtype-Ub and treated with MG132, and the IP samples and whole cell lysates were analyzed by immunoblotting. **F** IP samples and whole cell lysates of HEK293T cells transfected with Flag-Skp2 and Myc-SLC3A2 without MG132 treatment were analyzed by immunoblotting. **G** The distribution of Skp2 and SLC3A2 in MLE-12 cells was detected by immunofluorescence staining, scale bar = 10 μm

As the regulatory function of Skp2 relies on its ability to transfer ubiquitin to binding substrates, we used immunoprecipitation combined with mass spectrometry (IP-MS) to identify potential substrates of Skp2 by utilizing MLE-12 cells overexpressing Skp2 (Fig. 5A). Among all the Skp2-binding proteins, SLC3A2 (also known as CD98hc or 4F2hc) (Supplemental Fig. 5A), a cell-surface transmembrane protein that functions upstream of ferroptosis [33], decreased after the onset of sepsis (Fig. 5B), suggesting that the decrease in SLC3A2 during sepsis may be mediated by Skp2. Furthermore, we observed that Skp2 can ubiquitinate SLC3A2 (Fig. 5C), indicating a potential relationship between SLC3A2 ubiquitination and Skp2. To validate this association, we investigated various types of ubiquitination in cells [34, 35] and employed a panel of ubiquitin mutants. Notably, the predominant poly-Ub forms were found to be K48 linked, as evidenced by the nearly abolished coimmunoprecipitation of a K48R Ub mutant with SLC3A2 compared to that of other mutants or wildtype Ub (Fig. 5D). Subsequently, we transfected cells with HA-K48-Ub, HA-K63-Ub, or wildtype Ub to validate the K48-linked ubiquitination of SLC3A2. Notably, positive results were obtained for both K48 Ub and wildtype Ub (Fig. 5E). Moreover, the ubiquitination of SLC3A2 (Fig. 5F) also occurred even in the absence of MG132, indicating that the regulatory effect of Skp2 on SLC3A2 did not rely on ubiquitin-mediated protein degradation. The K48 ubiquitination pathway did not function as a degradation mechanism in the present study [36].

Interestingly, there is crosstalk and interplay between ubiquitin and substrates, with an emphasis on substrate selectivity and regulation under cellular irritation [37]. Skp2 promoted Akt ubiquitination and activation, which are required for the mitochondrial localization of Akt [38]. The non-degradative mechanism employed by Skp2 regulates the ubiquitination of substrates during the cell cycle [39]. In the present study, we observed that the cellular localization of SLC3A2, which colocalized with Skp2 shifted from a dispersed intracellular distribution to an aggregated state on the cell membrane after RCM treatment (Fig. 5G), suggesting that Skp2 regulated SLC3A2 translocation under inflammatory conditions [40]. Therefore, we speculated that Skp2 promoted the ubiquitination of SLC3A2 via a K48-linked nondegradative mechanism and led to the translocation of SLC3A2.

### Skp2 deficiency triggered ferroptosis by inhibiting SLC3A2 ubiquitination and disrupting cystine/glutamate exchange

**Fig. 6:**
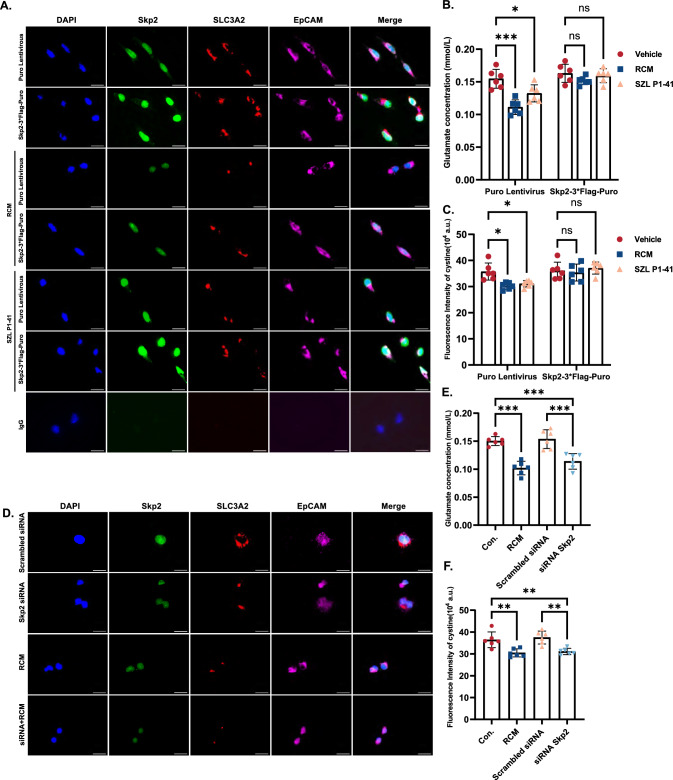
Skp2 deficiency triggered ferroptosis by inhibiting SLC3A2 ubiquitination and disrupting cystine/glutamate exchange. **A**–**C** MLE-12 mouse lung epithelial cells were transfected with Skp2-3*Flag-PGK-Puro to overexpress Skp2, and Puro Lentivirus transfected MLE-12 cells were used as controls. **A** EpCAM functions as an indicator of epithelial cells and was used to indicate the interplay between SLC3A2 and the alveolar epithelium. The distributions of Skp2, SLC3A2 and EpCAM were detected by immunofluorescence staining. Scale bar = 20 μm; **B**, **C** transfected MLE-12 cells were stimulated with RCM or Skp2 inhibitor SZL P1-41. **B** The glutamate content in the cell culture supernatant was analyzed with a glutamate assay kit. **C** Cystine uptake by MLE-12 cells was analyzed with a cystine uptake kit. **D**–**F** MLE-12 cells were transfected with Skp2 siRNA to knock down Skp2. Scrambled siRNA was used as a control. **D** The distributions of Skp2 and SLC3A2 were detected by immunofluorescence staining in MLE-12 cells transfected with Skp2 siRNA, scale bar = 20 μm; **E**, **F** transfected cells were stimulated with RCM or control medium (Con.). **E** The glutamate content in the medium of MLE-12 cells transfected with Skp2 siRNA and stimulated with RCM was analyzed by a glutamate assay kit; **F** the cystine uptake of MLE-12 cells transfected with Skp2 siRNA and stimulated with RCM was determined with a cystine uptake detection kit. The data are presented as the means ± standard deviations (ns *P* > 0.05, *P < 0.05, **P < 0.01, ***P < 0.001)

The distinct cellular distribution of SLC3A2 after RCM treatment suggested the potential impact of its intracellular localization on its function as a subunit of the cystine/glutamate exchanger (xCT), one of the three crucial pathways in ferroptosis [41]. The xCT complex consists of SLC3A2 and SLC7A11, which allows the extrusion of glutamate and the internalization of cystine [42]. Cystine then transforms into cysteine, which is used to synthesize GSH. Dysfunction of SLC3A2 results in the malfunction of xCT, consequently promoting ferroptosis. The overexpression of Skp2 was found to reduce RCM-induced SLC3A2 aggregation (Fig. 6A). Skp2-overexpressing MLE-12 cells exhibited equivalent increases in glutamate discharge and cystine uptake after treatment with RCM (Fig. 6B, C). SZL P1-41 serves as a functional inhibitor of Skp2 by effectively binding to the F-box domain of Skp2, thereby preventing its association with Skp1 and the formation of the Skp2 SCF complex [43]. When cells were treated with SZL P1-41 which inhibited the interaction between Skp2 and SLC3A2, the membrane localization and exchange of SLC3A2 were significantly suppressed (Fig. 6A–C), elucidating the crucial functional significance of the ubiquitination of SLC3A2. In contrast, the distribution of SLC3A2 in Skp2-knockdown MLE-12 cells remained comparable to that observed after RCM treatment (Fig. 6D). Furthermore, the inhibition of Skp2 resulted in a significant decrease in glutamate release (Fig. 6E) and cystine uptake (Fig. 6F). These results suggested that Skp2 regulated the localization and functional capacity of SLC3A2 via ubiquitination, thereby regulating the transport of cystine and glutamate and resulting in intracellular lipid peroxidation. Therefore, the suppressed expression of Skp2 in sepsis patients hampered SLC3A2 ubiquitination and the translocation of cystine and glutamate, ultimately leading to ferroptosis.

### Skp2 overexpression improves sepsis-induced acute lung injury

**Fig. 7:**
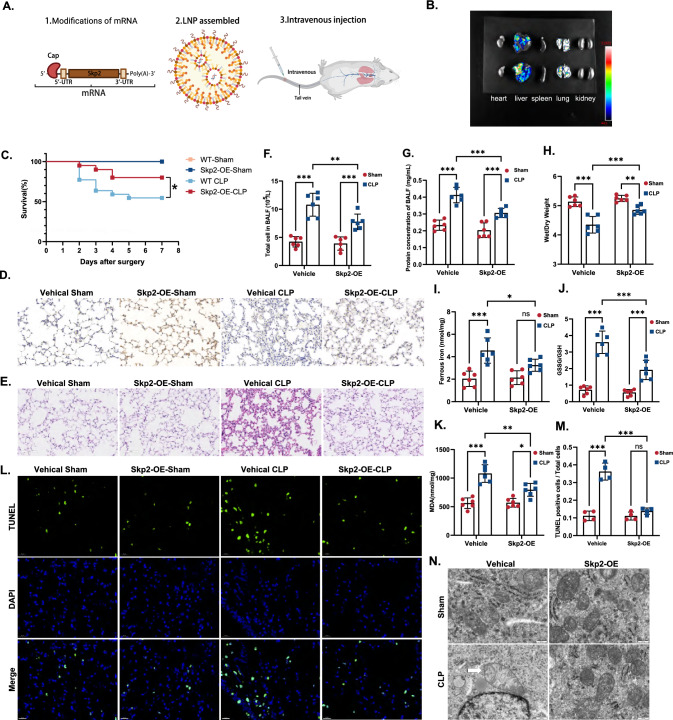
Skp2 overexpression improves sepsis-induced acute lung injury. Mice were assigned to Vehicle-Sham group, Vehicle-CLP group, Skp2-OE-sham (LNP injected) group or Skp2-OE-CLP (LNP injected) group. For LNP mice, lipid nanoparticles (LNPs) carrying Skp2 mRNA were injected via the tail vein. **A** Schematic diagram of LNP assembly (generated by Biorender). **B** After administering LNPs via intravenous injection, luciferase intensity in the heart, liver, spleen, lung and kidney was measured. **C** Survival analysis of each group, n = 24. **D**, **E** Histochemical staining (scale bar = 50 μm) and H&E staining (scale bar = 100 μm) of lungs were used to evaluate lung injury. **F** Cell counts in BALF of each group. **G** Protein concentration in BALF (mg/mL) of mice in each group. **H** Wet/dry ratio of lung tissues in each group. **I** Examination of the Fe^2+^ levels in lungs. **J** Lung GSSG/GSH ratio. **K** Lipid peroxidation as measured by the MDA concentration in the lungs. **L** TUNEL staining of the lungs of mice in each group, scale bar = 20 μm. **M** Quantification of TUNEL-positive cells among the total cell in each group. **N** Electron microscopy was used to visualize the ultrastructure of pulmonary mitochondria, scale bar = 500 μm. The data are presented as the means ± standard deviations (ns *P* > 0.05, *P < 0.05, **P < 0.01, ***P < 0.001)

To explore the therapeutic potential of Skp2 in septic lung injury, we developed lipid nanoparticles (LNPs) that encapsulate Skp2 mRNA, including its Cap1 structure, Skp2 domain, and poly(A) tail (Fig. 7A). These LNPs were administered via tail vein injection after the mice underwent CLP surgery to overexpress Skp2. After the administration of Skp2-loaded LNPs, Skp2 was successfully expressed in the lungs (Fig. 7B, Supplemental Fig. 6B). The liver exhibited high LNP expression, which was attributed to the preferential accumulation of cationic LNPs in the lungs and liver. As the primary blood-clearing organ, the liver possesses a significant capacity for LNP uptake [44]. More importantly, mice injected with LNPs exhibited reduced mortality (Fig. 7C) and ameliorated lung injury (Fig. 7D, E). Significant improvements in lung tissue injury after the administration of LNPs were further evidenced by reduced cell counts and total protein concentrations in the BALF, as well as reductions in the lung W/D weight ratio (Fig. 7F–H). The degree of ferroptosis in pulmonary epithelial cells was also significantly reduced after LNP infusion, as indicated by the decreased ferrous ion concentration (Fig. 7I), GSSG/GSH ratio (Fig. 7J), and MDA content (Fig. 7K). The reduction in lung injury was further supported by decreased epithelial cell death in the lungs as shown by TUNEL staining (Fig. 7L, M) and reduced mitochondrial damage (Fig. 7N). These data suggest that overexpressing Skp2 is effective in ameliorating sepsis-induced ALI and highlight the potential of LNPs carrying Skp2 mRNA for the treatment of sepsis.

## Discussion

Despite the lack of direct evidence, there are hints suggesting that Skp2 might function as a protective protein in sepsis-induced ALI. As reported in the literature, Skp2 was significantly inhibited during LPS-induced myocardial dysfunction [45], and the upregulation of circEXOC5 during acute lung injury accelerated Skp2 mRNA decay and aggravated sepsis-induced ALI. Consistently, we confirmed the association between Skp2 depletion and the occurrence of sepsis in the present study, showing that the repression of Skp2 is implicated in the progression of sepsis through the regulation of ferroptosis. Obtaining epithelial cells from BALF, especially from healthy donors, can be challenging due to the typically low cell yield. However, BALF provides a valuable and ethically feasible source for studying lung pathology. This allows us to investigate the expression of Skp2 in lung tissue, albeit indirectly, while also addressing the practical and ethical challenges associated with direct lung tissue research. Based on the results of BALF, lung tissue, and pulmonary epithelial cell experiments, we found that Skp2 suppression during sepsis causes changes in the oxidative-reductive system in pulmonary epithelial cells, leading to ferroptosis. Although homozygous knockouts offer robust evidence for causality due to comprehensive gene elimination among individuals, it should be noted that these genetically altered animals occasionally manifest severe phenotypes. Conversely, heterozygous knockouts maintain one functional copy of the target gene, potentially enhancing mouse health and longevity without compromising the intended genetic modification [46]. Notably, in our study, Skp2 heterozygous knockout mice displayed a more pronounced lung injury phenotype during sepsis than did wildtype mice. Skp2-mediated ubiquitination of the ferroptosis regulatory protein SLC3A2 could significantly influence its ability to regulate the transport of substances across the cell membrane. This function reveals the unconventional role of the ubiquitination system in the context of lung injury and ferroptosis. Enhancing our understanding of Skp2 could provide insights into the details of ubiquitination and reveal specific mechanisms that regulate protein homeostasis and signal attenuation in biological systems. More importantly, we developed an efficient way to overexpress Skp2 in the lungs of mice and provided evidence that targeting Skp2 could be a potential strategy for treating sepsis-induced ALI through ferroptosis intervention.

In addition to ferroptosis, other types of cell death are also induced by inflammatory cytokine storms in sepsis. To determine whether the decreased expression of Skp2 is related to these processes, we observed the expression of Skp2 in mice or cells undergoing ferroptosis, pyroptosis, and apoptosis (Supplemental Fig. 3A–E). However, when pyroptosis was induced by LPS + CTB [47] or apoptosis was induced by procaspase activating compound-1 (PAC-1) [48], we did not detect lipid oxidation, which is characteristic of lung epithelial cells undergoing ferroptosis induced by Erastin (Supplemental Fig. 3D). In addition, the pan-caspase inhibitor Z-VAD-FMK [49] and the RIP1K inhibitor necrostatin-1 (Nec-1) [50] (Supplemental Fig. 3E) did not prevent the Erastin-induced downregulation of Skp2.

Moreover, the treatment with Fer-1 [51] led to a noticeable increase in the expression of Skp2 in the lungs of CLP mice compared to those in the control group (Supplemental Fig. 4A). Fer-1 attenuated the inhibitory effect of Erastin on Skp2 and GPX4 (Supplemental Fig. 4B). These data suggest that Skp2 downregulation occurs during ferroptosis. Taken together, these data indicate that the inhibition of Skp2 specifically affects ferroptosis of lung epithelium.

Given the intricate mechanism underlying sepsis-induced death of pulmonary epithelial cells [52], we confirmed the specificity of decreased Skp2 expression in ferroptosis. However, it is unclear whether the decreased expression of Skp2 was the cause or the consequence of ferroptosis. The downregulation of Skp2 could be attributed to its involvement in the cascade of inflammatory reactions and cell death. A complex interplay exists between cytokines and chemokines in the context of inflammatory microenvironments [53, 54]. The MEK-ERK kinase pathway transmits signals from cell surface receptors to transcription factors, thereby regulating gene expression. Depending on the stimulus and cell type, this pathway can either inhibit or induce programmed cell death [55]. Ferroptosis, which has been experimentally linked to MEK-dependent oxidative cell death, can be effectively attenuated by the administration of MEK inhibitors [56]. The MEK-ERK kinase pathway is a commonly activated in response to infection and cell or tissue damage [57]. Moreover, Erastin also facilitated the inhibition of Skp2 via activation of the MEK-ERK kinase pathway (Supplemental Fig. 4C). The association of the MEK-ERK kinase pathway with Skp2 provides mechanistic insight into the cascade of inflammation activation, alteration of target proteins (Skp2 inhibition), and ferroptosis. Through these experiments, we demonstrated the intricate association between Skp2 and ferroptosis in sepsis. However, our study focused on the complex system by which inflammatory factors trigger alveolar epithelial cell death without exploring the specific contributions of individual factors. Future studies could focus on the assessment of various combinations of cytokines to determine their respective roles.

The present study further indicated that Skp2 modulates ferroptosis via ubiquitination. To identify the ubiquitinated substrate of Skp2 and the potential targets involved in ferroptosis, IP-MS was proven to be a reliable approach [58]. The Skp2-binding protein SLC3A2 contributes to the modulation of ferroptosis via its interaction with SLC7A11 to sustain the transport of cystine and glutamate [59]. However, a pronounced decrease in the expression of both Skp2 and SLC3A2 was observed in the lungs of septic mice, which contradicts the classical function of ubiquitination [60]. Interestingly, SLC3A2 ubiquitination was observed even in the absence of MG132-mediated proteasome inhibition, suggesting that K48-linked SLC3A2 ubiquitination is a possible mechanism of signal transduction that might not be exclusively linked to protein degradation. SZL P1-41 has been shown to impede substrate ubiquitination by interacting with Skp2. By utilizing immunofluorescence to observe the intracellular distribution of SLC3A2 and measuring material transport to verify its function, we revealed the intricate interplay between SLC3A2 and the process of ubiquitination and revealed a novel mechanism by which Skp2 and SLC3A2 function in sepsis-mediated ferroptosis.

The complexity of the ubiquitination system arises from the various topologies of ubiquitin chains on substrates [61], which confer upon this system the ability to modulate many signaling pathways that underlie cellular functions [62]. The precise impact of K48 ubiquitination on the function of SLC3A2 remains unclear. This effect may occur via an unconventional ubiquitination chain structure [63] that influences the function of SLC3A2. A previous study proposed that the conformation of the K48 ubiquitin chain comprises cyclization and noncyclization. Cyclized ubiquitin chains affect substrate function due to abnormal conformational changes within binding regions [64]. K48 ubiquitination in combination with other branches could either enhance or suppress its original effect [65]. E3 ubiquitin ligases are capable of mediating K11/K48- [66], K29/K48- [67], and K48/K63-branched chains [68]. Branching might have an important impact on the maintenance of ubiquitin signaling [69]. The significance of ubiquitination as a cellular signal that regulates changes in the cellular environment is indicated by its spatial and temporal dynamics [70]. Currently, there is a lack of clarity regarding the atypical ubiquitin modification of Skp2 substrates. It is imperative to elucidate the precise regulatory effects of the ubiquitination system on SLC3A2 through topological methods in upcoming studies.

Current treatments for sepsis predominantly target immune cells [71] and inflammatory factors [72], but the efficacy of these interventions has been consistently unsatisfactory. Therefore, the delivery of mRNA to the lungs is a viable alternative strategy for mitigating the consequences of absent or defective Skp2 function and potentially alleviating the impairment of lung function [73]. LNPs are an established delivery system for mRNA therapeutics, and they have strong potential for use as an efficacious treatment approach in the future [74]. Upon cellular uptake in the lungs [75], the delivered Skp2 mRNA is subsequently released into the cytoplasm and efficiently translated, consequently restoring the functional integrity of membrane transport in alveolar epithelial cells. To date, the efficacy of LNP technology has been enhanced through the regulation of lipid composition and content, while transfection efficiency has improved through the implementation of novel techniques [76]. These LNPs satisfy all the requisite conditions for successful delivery systems, including specific Skp2 mRNA modification patterns, proportionate lipids, and the ability to reassemble. The scalable and nonviral characteristics of LNPs present promising potential for serving as a clinically viable therapy for sepsis-induced ALI.

In conclusion, we identified a distinct mechanism by which ferroptosis is regulated by ubiquitination through an extensive investigation of sepsis-induced acute lung injury. Skp2, which functions as an E3 ubiquitin ligase, exhibited noteworthy anti-inflammatory effects on ferroptosis triggered by inflammatory cytokines, and represents a promising approach for the management of sepsis-induced ALI.

## Methods

### Key resources table

**Table:** 

Reagent or resource	Source	Identifier
Antibodies		
Skp2 polyclonal antibody	Proteintech	15010-1-AP
Anti-glutathione peroxidase 4 antibody	Abcam	ab125066
Goat anti-rabbit IgG-HRP	Absin	abs20002
Anti-mouse IgG, HRP-linked antibody	Cell Signaling Technology	Anti-mouse IgG, HRP-linked Antibody
GSDMD antibody	Santa cruz	sc-393581
MEK1/2 antibody	Cell Signaling Technology	#9122
Phospho-MEK1/2 (Ser217/221)	Cell Signaling Technology	#9154
p38 MAPK antibody	Cell Signaling Technology	#9212
Phospho-p38 MAPK (Thr180/Tyr182)	Cell Signaling Technology	#9211
SAPK/JNK antibody	Cell Signaling Technology	#9252
Phospho-SAPK/JNK (Thr183/Tyr185)	Cell Signaling Technology	#9251
p44/42 MAPK (Erk1/2)	Cell Signaling Technology	#4695
Phospho-p44/42 MAPK (Erk1/2)	Cell Signaling Technology	#4370
Chemicals, peptides, and recombinant proteins		
RIPA buffer	Thermo Fisher	89900
LPS	Sigma-Aldrich	L2880
Pierce^TM^BCA Protein Assay Kit	Thermo Fisher	23225
Dulbecco modified eagle medium (DME)/Ham Nutrient Mixture F-12	Sigma-Aldrich	D9785
StableCell™ DMEM	Sigma-Aldrich	D0822
DAPI Fluoromount-G^®^	SouthernBiotech	0100-20
FICOLL PAQUE PLUS	Cytiva	17144002
RBC lysis buffer (10 ×)	Biolegend	420301
IMMOBILON WESTERN CHEMILUM HRP SUBSTRATE	Millipore	WBKLS0500
PrimeScript™ RT Master Mix	Takara	RR036
TB Green^®^ Premix Ex Taq™ II	Takara	RR820
PageRuler Plus Prestained Protein Ladder	Thermo Fisher	26620
DMSO	Sigma-Aldrich	C6164
Immobilon^®^-FL PVDF	Millipore	IPFL00010
Critical commercial assays		
Iron Assay Kit	Abcam	ab83366
Lipid Peroxidation (MDA) Assay Kit	Abcam	ab233471
Cystine Uptake Assay Kit	DOJINDO	UP05
Glutamate Assay Kit-WST	DOJINDO	G269
Bodipy™ 581/591 C11	Invitrogen	D3861
BD Pharmingen™ FITC Annexin V Apoptosis Detection Kit	BD	556547
Medicines		
Lipopolysaccharide	Sigma‒Aldrich	L5293
Erastin	Sigma-Aldrich	E7781
Ferrostatin-1	MedChemExpress	HY-100579
SMIP004	MedChemExpress	HY-15694
PAC-1	MedChemExpress	HY-13523
Z-VAD-FMK	MedChemExpress	HY-16658B
Necrostatin-1	MedChemExpress	HY-15760
AZD8330	MedChemExpress	HY-12058
SZL P1-41	MedChemExpress	HY-100237
FR180204	MedChemExpress	HY-12275
C16-PAF	MedChemExpress	HY-108635
(rel)-AR234960	MedChemExpress	HY-120006A
SP600125	Selleck Chemicals	S1460
SB203580	Selleck Chemicals	S1076

## Method details

### Ethics statement

This study was approved by the Ethics Committee of Zhongshan Hospital of Fudan University. The protocol adhered to the principles outlined in the Declaration of Helsinki. Prior to participation, either the patients themselves or their respective relatives provided written informed consent (Protocol license number: B2021-182R). All animal experiments were carried out in compliance with the guidelines of the Animal Review Committee at Zhongshan Hospital of Fudan University (Protocol license number: 2020-119).

### Patients

Biological samples were prospectively collected from 6 patients with sepsis in Zhongshan Hospital of Fudan University from January 2022 to March 2022. Moreover, general data and various examination data of patients were collected. Patients aged 18–80 years who met the Sepsis 3.0 guidelines [77] and ARDS Berlin criteria [78] were included. Exclusion criteria included malignancy, autoimmune disorders, acute cardiovascular and cerebrovascular ailments, a history of intensive care unit treatment, active massive hemorrhage, recent blood transfusion within two weeks, and pregnancy or lactation in females. Six healthy donors were enrolled in this study, all of whom were admitted for evaluation of solitary pulmonary nodules without any evidence of pulmonary infection. Informed consent was obtained from all individual participants included in the study. Bronchoalveolar lavage fluid (BALF) was performed and cells in BALF were collected for analysis.

### Isolation of BALF cells

A total of 20 mL of BALF was collected and promptly placed on ice. The BALF was then filtered through a 40 µm nylon cell strainer to remove clumps and debris. Following filtration, the resulting supernatant was centrifuged, and the cells were resuspended in RPMI 1640. The BALF obtained from patients and healthy controls exhibited cell concentrations ranging from 0.5–15 × 10^4^/mL. The cells were subsequently counted and resuspended at a concentration of 10^6^/mL for further experimental procedures. Approximately 5% of epithelial cells were identified within these groups.

### Cecal ligation and puncture (CLP) mouse model

C57BL/6 mice (22–25 g, 8–10 weeks, male) were purchased from Shanghai Lab. Animal Research Center. All the animals were housed in a specific pathogen-free facility; the room was maintained at a constant temperature of 22 ± 2 °C. Age- and sex-matched wildtype and Skp2^+/−^ mice were intraperitoneally injected with 2% pentobarbital sodium for anesthesia. The skin and muscle layers were meticulously separated, revealing the cecum. Using a 4–0 silk thread, ligation was performed two-thirds of the way from the end of the cecum, followed by distal penetration of the cecum using a 22 G needle. Muscles and skin were closed with suture. An initial dose of 1 mL of warmed normal saline was administered immediately after the surgical procedure. To alleviate pain and discomfort, buprenorphine (0.05 mg/kg) was administered subcutaneously for analgesia. The mice were kept on a 37 °C warming pad to sustain their body temperature until they regained consciousness. Antibiotic treatment with imipenem (25 mg/kg) was initiated 2 h after CLP. The model was established according to the Minimum Quality Threshold in Pre-Clinical Sepsis Studies (MQTiPSS) [79]. Mice in Sham group underwent a sham laparotomy. Humane endpoints were utilized to determine whether the mice were in a moribund state and met the criteria for euthanasia. These criteria included the inability to stand, agonal breathing, a decrease in body temperature to less than 30 °C for more than 6 h, or excessive loss of body weight.

### Survival assays

The mortality rates of the experimental mice in the groups were recorded daily until the end of the seventh day. There were 24 mice in each group.

### Cell cultures

The murine macrophage line Raw264.7, murine alveolar macrophage cell line MH-S, and lung epithelial cell line MLE-12 were purchased from National Collection of Authenticated Cell Cultures. Raw264.7 cells were plated in 10 cm petri dishes at a density of 10^5^ cells/mL. Once the cells had attached to the well, 100 ng/mL LPS was added to stimulate the Raw264.7 cells. After 12 h, the Raw264.7 cells started to differentiate, shifting from adherent spheres to polygons and developing pseudopods. The supernatants were subsequently obtained and were subjected to centrifugation at a speed of 2000 rpm for a duration of 5 min. Subsequently, the supernatants were carefully added to a six-well plate with MLE-12 cells for 4 h. Raw 264.7 were kept in 10% FBS/Cell Culture DMEM. A mixture of DMEM/F12 containing 10% FBS was used to maintain MLE-12 cells. MH-S cells were cultured in 10% FBS/RPMI-1640.

### Lentiviral transfection

A Skp2 overexpressing lentiviral virus was purchased from Genomeditech (gene ID: NM_013787.3, vector: PGMLV-CMV-MCS-3 × FLAG-PGK-Puro). HEK293T cells were incubated overnight, resulting in a cell density of approximately 30–50%. Next, the virus was diluted to an MOI of 50 and added to the cells overnight. The culture medium was replaced with puromycin-supplemented medium at a concentration of 8 μg/mL, and the cells were subsequently maintained for 72 h. The expression of Skp2 was measured using quantitative real-time PCR and western blotting to assess the efficiency of Skp2 overexpression.

Skp2 siRNA was purchased from Obiosh, and the sequence of Skp2 siRNA used were 5′-GGGCAAAGGGAGUGACAAATT-3′ and 5′-UUUGUCACUCCCUUUGCCCTT-3′. The siRNA was diluted in Opti-MEM^®^ medium, followed by the addition of transfection reagent after proper mixing by vortexing and incubation. The mixture was transferred to MLE-12 cells after the medium was changed. The cells were incubated for 48 h, followed by quantification of the mRNA and protein levels at 72 h posttransfection.

### Quantitative real-time polymerase chain reaction (qPCR)

Cells in BALF were treated with an RNA extraction kit (Yishan Biotechnology), the RNA was reverse transcribed into cDNA (TaKaRa, Japan), and the target gene was amplified by PCR (TaKaRa, Japan).

The primers used were as follows: Skp2 forward 5′-AGTCTCTATGGCAGACCTTAGACC-3′, reverse 5′TTTCTGGAGATTCTTTCTGTAGCC-3′; β-actin forward 5′-AAGGTGACAGCAGTCGGTT-3′, reverse 5′-TGTGTGGACTTGGGAGAGG-3′

### Immunoblotting

A total of 30–60 μg of protein per lane was subjected to SDS‒PAGE with constant electrophoresis at 90 V until the complete separation of proteins with varying molecular weights was achieved. The proteins were subsequently transferred to PVDF membranes. The bands were developed and analyzed after incubation with primary antibodies and secondary antibodies. Further details about the antibodies used are listed in the key resources table.

### Cell slide immunofluorescence

The cells were transferred to a lysine-coated coverslip and plated in a 24-well plate. Following adequate processing and stimulation, the cells were gently rinsed with PBS. The cells were treated with 4% paraformaldehyde (PFA) for 15 min at room temperature. Membrane permeabilization was performed by treatment with 0.05% Triton X-100. Subsequently, the cells were incubated with 3% bovine serum albumin (BSA) in PBS to minimize background staining. After incubation with the primary and secondary antibodies, the slides were sealed with a fluorescence quenching agent containing DAPI. The protocol for triple fluorescence staining was the same as before, up to the step of applying the primary antibody. After overnight incubation with the primary antibody at 4 °C, the corresponding HRP-conjugated secondary antibody was added and the membrane was allowed to incubate. Subsequently, 50–100 μL of TSA-488/555/594 staining working solution was applied to the tissue, ensuring complete coverage. The sample was then incubated at room temperature in the dark for 10 min, and finally sealed with an anti-fluorescence quenching mounting agent. The average fluorescence intensity (mean) was analyzed using Fiji software, where mean was calculated as the total fluorescence intensity (IntDen) divided by the area of the corresponding region (area).

### HE staining

The sections underwent were dewaxed by immersion in xylene and ethanol at varying concentrations, followed by hematoxylin staining, differentiation, and washing. Subsequently, eosin dye solution was used for staining, and dehydration was performed before the sections were sealed in sequential ethanol baths with differing concentrations.

### Electron microscopy

Lung tissues obtained from mice were sectioned into small pieces. The tissue was fixed in a suitable fixative solution, specifically 2.5% glutaraldehyde in 0.1 M phosphate buffer. Subsequently, the samples were incubated in an electron microscope fixation solution, and then subjected to further incubation in 1% osmic acid for 1–2 h at room temperature. The dehydrating process was carried out using varying concentrations of alcohol, with 20-min incubations at each concentration. The tissues were continuously incubated in 100% acetone, with each incubation lasting 15 min. Following embedding, the plate was incubated in a 60 °C temperature-controlled box for 48 h, after which the resin block was sliced using a microtome. Subsequently, the sections were stained with uranium acetate saturated alcohol solution and lead citrate solution to enhance contrast. After staining, the sections were carefully cleaned, allowed to dry overnight, and subsequently analyzed using a transmission electron microscope (TEM) at an appropriate magnification.

### TUNEL staining

The frozen sections were thawed at 37 °C to eliminate moisture before being fixed with 4% paraformaldehyde for 30 min. To facilitate antigen repair, protease K was added, and the samples were incubated. Subsequently, a membrane permeabilization working solution was added and incubated. A mixture of the TDT enzyme, dUTP, and buffer was then prepared according to the instructions and added to the tissues. DAPI was used to stain the nuclei in the dark. The sections were sealed and imaged under a microscope.

### Lung wet-to-dry ratio

The procedure involved dissecting and harvesting the right lung, followed by measuring its weight (wet weight). Subsequently, the lung was dried overnight at 60 °C to obtain the dry weight. The wet weight/dry weight ratio was calculated by dividing the wet weight by the dry weight.

### Cell count and protein concentration in BALF

A small incision was made in the neck to expose the mouse trachea. A cannula was inserted into the trachea and securely fixed in place. A gentle instillation of 1 mL of sterile saline into the lung was performed through the cannula. The fluid was then aspirated back into the syringe, ensuring complete recovery. The BALF was transferred to a sterile tube and centrifuged to pellet the cells. The supernatant was removed for protein concentration analysis using the BCA assay. The cells were resuspended in PBS, and cell counting was conducted using a hemocytometer.

### Quantification of cellular GSSG/GSH levels

Ten milligrams of mouse lung tissue was weighed and washed in precooled PBS. A GSSG/GSH Quantification Kit II (G263) was used according to the instructions of Dojindo. The absorbance was measured with a microplate system, and the GSH and GSSG concentrations were calculated.

### Malondialdehyde (MDA) quantification

First, 10 mg of mouse lung tissue was weighed and washed with precooled PBS before preparing the lysis solution. Ultrasonic cell disruption may be utilized for lysis, followed by subsequent centrifugation at a rate of 13,000*g*/10 min to glean the resulting supernatant. A standard lipid curve was then prepared, along with the necessary sample formulations, following the recommended protocol for Abcam lipid peroxidation (MDA) assay (ab118970).

### Concentration of ferrous ions

The content of ferrous iron was determined using an Abcam iron assay kit (ab83366). Ten milligrams of lung tissue was weighed and washed with cold PBS. Iron assay buffer was added, and the tissue was homogenized at 16,000 g for 10 min. Gradient concentrations of standard and test samples were then added to a 96-well plate, and assay buffer and probe were added to each well. The absorbance was measured at OD593 nm, and concentration of ferrous ions in the test samples was calculated.

### Analysis of lipid peroxidation

For flow cytometry analysis, a Bodipy probe (Invitrogen™ D3861) was added to the cell medium at a final concentration of 2.5 µM. The cells were then collected and digested with pancreatic enzymes, and confirmed by the flow cytometry.

Lung epithelial cells were treated with confocal glass bottom dishes for immunofluorescence using fluorescence staining. Bodipy probe was added at a final concentration of 5 μM. Immunoprecipitation and mass spectrometry were then performed, with fluorescence imaging carried out using 488 nm and 594 nm microscopic wavelengths, after washing with PBS.

### Apoptosis analysis

Treated MLE-12 cells were dissociated using EDTA-free pancreatic enzymes. Thereafter, 300 μL of binding buffer was added to tubes, followed by the addition of 5 μL of Annexin V-FITC. The cells were thoroughly mixed and incubated for 15 min. Finally, 5 μL of PI was added for staining, followed by a 5-min suspension prior to machine analysis.

### Immunoprecipitation mass spectrometry

IP-MS cell lysis buffer containing PMSF was added to a Petri dish, mixed thoroughly, and subsequently incubated on ice for a period of 20 min. The supernatant was collected, and an anti-Flag antibody was added to the supernatant overnight. Then, protein A/G magnetic beads were introduced into EP tubes containing the aforementioned antibodies and subsequently incubated overnight at 4 °C. IP-MS wash buffer was used to wash the magnetic beads. Subsequently, loading buffer was added to the EP tube and incubated in a metal bath at 100 °C for 10 min. A magnetic rack enabled the separation of the magnetic beads and subsequent absorption of the supernatant liquid containing the target protein. After electrophoresis, the proteins were silver stained, the protein bands were excised, and the samples were subjected to mass spectrometry analysis.

### Ubiquitination experiment

The following plasmids were purchased from Addgene: HA-Ub (17608), HA-Ub-K48 (17605), HA-Ub-K48R (17604), and HA-Ub-K63 (17606). Myc-SLC3A2, Flag-Skp2 and other ubiquitin mutants were obtained from Zhizhang Wang (Department of Pathology, Nanfang Hospital, Southern Medical University, Guangzhou, Guangdong, China). In accordance with the experimental aim, 20 μM MG-132 was administered or not to the cells. Prechilled lysis buffer containing protease inhibitor was used for cell lysis and collection of the supernatant. A portion of the whole-cell lysate was retained as a control, while the rest of the protein lysate was incubated overnight with 20 µL of beads. Then, 100 μL of loading buffer was added, and the samples were incubated at 100 °C and frozen. The Western blot protocol was followed. The concentration of the separation gel that was used corresponded to the molecular weight of the target protein. A corresponding labeled antibody was used to detect the target protein, and an anti-HA antibody was used to detect ubiquitination of the target protein.

### Cystine absorption experiment

Cystine absorption experiment was determined using Dojindo Cystine Uptake Assay Kit (UP05). The black 96-well plates were seeded with cells and incubated overnight. The cystine-free medium was replaced with medium containing Cystine Analog, and the cells were incubated at 37 °C for 30 min. Then, 50 μL methanol was added to each well, 200 μL of the prepared working solution was added, and the plates were sealed to prevent evaporation. The plates were incubated for 30 min. A microplate reader with fluorescence detection capabilities was utilized to measure the fluorescence intensity of the labeled cells. The fluorescence intensity was assessed at an excitation wavelength of 490 nm and an emission wavelength of 535 nm. To determine the specific fluorescence intensity resulting from the cellular uptake of the cystine analog, the values measured in the sample wells were subtracted from the values detected in the blank wells.

### Glutamate concentration measurement

Glutamate concentration was measured using Dojindo Glutamate Assay Kit (G269). The 96-well plates were seeded with cells and incubated overnight. Serum-free medium without cystine was warmed to 37 °C and added to the wells. Next, cystine medium was added at 37 °C for 30 min, followed by the addition of three washes with cold PBS. Methanol (50 μL) was added to each well, followed by 200 μL of the prepared working liquid. The wells were sealed with sealing plate film to prevent evaporation of the liquid and incubated for 30 min. The fluorescence intensity of each well was measured using a fluorescence spectrophotometer at wavelengths of 490 nm and 535 nm, and the results were subsequently calculated.

### Synthesis of LNP particles

Skp2 mRNA (NM_013787.3, Consensus CDS: CCDS37037.1) was synthesized by ApexBio Technology (China). The synthesized mRNA was dissolved in 1 mM sodium citrate (pH 6.4) at 1388.9 ng/μL and A260/280 = 2.08.

The components of the lipid nanoparticles were dissolved in 100% ethanol. The molar ratio of the LNP components ionizable lipids/DSPC/cholesterol/DMG-PEG2000 was 50/10/38.5/1.5. The mRNA was dissolved in a 50 mM acetic acid buffer (pH 4.5), and the aqueous phase (mRNA) and organic phase (LNP component) were mixed by Precision Nanosystems nanoassembly at a 3:1 ratio. The LNPs were collected and diluted in PBS (pH 7.2). A 10 kDa Slide a-Lyzer G2 Dialysis Cassette was used to dialyze the LNPs overnight at 4 °C. After filtering through a 0.2 mm sterile filter, the final RNA load concentration was 598 ng/μL. The LNPs were stored at 2 °C to 8 °C. The particle size and polydispersity of LNPS were measured by a Malvern Zetasizer dynamic light scattering (DLS) instrument. RNA packaging efficiency was measured with a Quant-it RiboGreen RNA assay kit.

### Statistical analysis

Statistical analysis and graphing of the data were conducted using GraphPad Prism 9.5.1 software. All the data are presented as the mean ± SD, and the experimental sample size and values are indicated using individual data points. Before conducting the comparisons, the normality of the distributions was assessed using the Shapiro–Wilk test. Differences between two groups were analyzed using Student’s t test. Single-factor data analysis utilized one-way ANOVA multiple comparisons test, while two-factor data analysis utilized two-way ANOVA for multiple comparisons. Survival analysis was performed through the use of Kaplan‒Meier curves. Significance was defined as P < 0.05. P-values and sample sizes are provided in the main text as well as in the legends of the supplementary figures.

### Supplementary Information

Below is the link to the electronic supplementary material.

Supplementary file1 Figure S1. The inflammatory cytokine storm inhibited Skp2 expression. (A) Skp2 protein expression in murine lungs at 12, 24 and 48 hours after CLP was measured by Western blotting; (B) MLE-12 cells were stimulated with RCM for varying time intervals (0.5, 1, 2, 3, and 4 hours), and Skp2 protein expression levels were measured by Western blotting; (C) The concentrations of cytokines in RCM were quantified using the Cytometric Bead Array (CBA). (D-I) Statistical analysis was conducted to determine the concentrations of IL-6, TNF-α, MCP-1, IL-10, IL-12, and IFN-γ in the supernatants of Raw264.7 cells treated with control or LPS (RCM). (J) MLE-12 cells were stimulated with 300 ng/mL TNF-α and 10 ng/mL IFN-γ, and Skp2 protein levels in each group were measured by western blotting. The data are presented as the means ± standard deviations (ns p>0.05, *P<0.05, **P<0.01, ***P<0.001). Figure S2. The inhibition of Skp2 exacerbated ferroptosis in the lung epithelium. (A-B) MLE-12 lung epithelial cells were transfected with Skp2 lentivirus (Skp2-3*Flag-PGK-Puro) or control lentivirus and selected with puromycin. The protein expression levels of Skp2 and GPX4 were measured by western blotting after lentivirus transfection and treatment with RCM(A) or Erastin (B). (C-D) Skp2 in MLE-12 cells was knocked down by siRNA(C) or inhibited by Skp2 inhibitor SMIP004(D), and the cells were treated with RCM. The protein expression levels of Skp2 and GPX4 in MLE-12 cells were measured by western blotting. Lipid peroxidation was analyzed by Bodipy fluorescence by flow cytometry after Erastin (E) or SMIP004 (F) treatment. The data are presented as the means ± standard deviations (ns P>0.05, *P<0.05, **P<0.01, ***P<0.001). Figure S3. Skp2 inhibition mainly occurs during ferroptosis. (A) Pyroptosis of MLE-12 cells was induced by LPS+CTB, and the expression of Skp2, GPX4 as and the N-terminal protein GSDMD was measured by western blotting. (B-C) The procaspase-3 activator PAC-1 was used to induce MLE-12 cell apoptosis. Annexin V+PI+ cells were then detected by flow cytometry to analyze the degree of cell apoptosis. (D) Cells were transfected with the lipid peroxidation probe Bodipy and analyzed by flow cytometry (# Compared with control group，P>0.05, **P<0.01). (E) MLE-12 cells were treated with Erastin, the pancaspase inhibitor V-VAD-FMK, and Necrostatin-1. The expression levels of Skp2 and GPX4 in MLE-12 were then measured by western blotting. The data are presented as means ± standard deviations (ns P >0.05, *P<0.05, **P<0.01, ***P<0.001). Figure S4. Fer-1 has been shown to mitigate the inhibitory effects of Skp2. (A) Wildtype sham or CLP mice were intravenously injected with ferrostatin-1 (Fer-1) (3 mg/kg) and Skp2 and GPX4 protein expression was measured by western blotting. (B) Skp2 and GPX4 protein expression was measured by western blotting analysis in MLE-12 treated with Erastin and/or Fer-1. (C) The expression levels of p-MEK and MEK were detected by western blotting after Erastin stimulation. The data are presented as means ± standard deviations (ns P>0.05, *P<0.05, **P<0.01, ***P<0.001). Figure S5. SLC3A2 is a Skp2-binding protein. (A) Anti-Flag was used for immunoprecipitation (IP) in MLE-12 cells, and the IP samples and whole cell lysates (input) were analyzed by immunoblotting. Figure S6. LNP Particle Characterization and Efficacy. (A) The LNP particle size was measured by dynamic light scattering (DLS) using a Malvern Zetasizer DLS instrument. (B）The expression levels of Skp2 and GPX4 in the lungs of mice in each group were measured by Western blotting. The data are presented as the means ± standard deviations (*P<0.05, ***P<0.001) (PDF 4399 KB)

Supplementary file1 (PDF 1562 kb)

## Data Availability

The datasets used and/or analyzed during the current study are available from the corresponding author upon reasonable request.

## References

[1] Kumar V (2020) Toll-like receptors in sepsis-associated cytokine storm and their endogenous negative regulators as future immunomodulatory targets. Int Immunopharmacol 89(Pt B):107087. 10.1016/j.intimp.2020.107087 33075714 10.1016/j.intimp.2020.107087 PMC7550173

[2] Chousterman BG, Swirski FK, Weber GF (2017) Cytokine storm and sepsis disease pathogenesis. Semin Immunopathol 39(5):517–528. 10.1007/s00281-017-0639-8 28555385 10.1007/s00281-017-0639-8

[3] Karki R, Kanneganti TD (2021) The ‘cytokine storm’: molecular mechanisms and therapeutic prospects. Trends Immunol 42(8):681–705. 10.1016/j.it.2021.06.001 34217595 10.1016/j.it.2021.06.001 PMC9310545

[4] Lei G, Zhuang L, Gan B (2022) Targeting ferroptosis as a vulnerability in cancer. Nat Rev Cancer 22(7):381–396. 10.1038/s41568-022-00459-0 35338310 10.1038/s41568-022-00459-0 PMC10243716

[5] Chen X, Kang R, Kroemer G, Tang D (2021) Ferroptosis in infection, inflammation, and immunity. J Exp Med. 10.1084/jem.20210518 33978684 10.1084/jem.20210518 PMC8126980

[6] Li J, Cao F, Yin HL, Huang ZJ, Lin ZT, Mao N, Sun B, Wang G (2020) Ferroptosis: past, present and future. Cell Death Dis 11(2):88. 10.1038/s41419-020-2298-2 32015325 10.1038/s41419-020-2298-2 PMC6997353

[7] Zhang H, Liu J, Zhou Y, Qu M, Wang Y, Guo K, Shen R, Sun Z, Cata JP, Yang S, Chen W, Miao C (2022) Neutrophil extracellular traps mediate m(6)A modification and regulates sepsis-associated acute lung injury by activating ferroptosis in alveolar epithelial cells. Int J Biol Sci 18(8):3337–3357. 10.7150/ijbs.69141 35637949 10.7150/ijbs.69141 PMC9134924

[8] Liao J, Su X, Wang M, Jiang L, Chen X, Liu Z, Tang G, Zhou L, Li H, Lv X, Yin J, Wang H, Wang Y (2023) The E3 ubiquitin ligase CHIP protects against sepsis-induced myocardial dysfunction by inhibiting NF-kappaB-mediated inflammation via promoting ubiquitination and degradation of karyopherin-alpha 2. Transl Res 255:50–65. 10.1016/j.trsl.2022.11.006 36400309 10.1016/j.trsl.2022.11.006

[9] Cockram PE, Kist M, Prakash S, Chen SH, Wertz IE, Vucic D (2021) Ubiquitination in the regulation of inflammatory cell death and cancer. Cell Death Differ 28(2):591–605. 10.1038/s41418-020-00708-5 33432113 10.1038/s41418-020-00708-5 PMC7798376

[10] Nguyen KT, Mun SH, Yang J, Lee J, Seok OH, Kim E, Kim D, An SY, Seo DY, Suh JY, Lee Y, Hwang CS (2022) The MARCHF6 E3 ubiquitin ligase acts as an NADPH sensor for the regulation of ferroptosis. Nat Cell Biol 24(8):1239–1251. 10.1038/s41556-022-00973-1 35941365 10.1038/s41556-022-00973-1

[11] Asmamaw MD, Liu Y, Zheng YC, Shi XJ, Liu HM (2020) Skp2 in the ubiquitin-proteasome system: a comprehensive review. Med Res Rev 40(5):1920–1949. 10.1002/med.21675 32391596 10.1002/med.21675

[12] Cai Z, Moten A, Peng D, Hsu CC, Pan BS, Manne R, Li HY, Lin HK (2020) The Skp2 pathway: a critical target for cancer therapy. Semin Cancer Biol 67(Pt 2):16–33. 10.1016/j.semcancer.2020.01.013 32014608 10.1016/j.semcancer.2020.01.013 PMC9201937

[13] Eygeris Y, Gupta M, Kim J, Sahay G (2022) Chemistry of lipid nanoparticles for RNA delivery. Acc Chem Res 55(1):2–12. 10.1021/acs.accounts.1c00544 34850635 10.1021/acs.accounts.1c00544

[14] Liao M, Liu Y, Yuan J, Wen Y, Xu G, Zhao J, Cheng L, Li J, Wang X, Wang F, Liu L, Amit I, Zhang S, Zhang Z (2020) Single-cell landscape of bronchoalveolar immune cells in patients with COVID-19. Nat Med 26(6):842–844. 10.1038/s41591-020-0901-9 32398875 10.1038/s41591-020-0901-9

[15] Couetil LL, Thompson CA (2020) Airway diagnostics: bronchoalveolar lavage, tracheal wash, and pleural fluid. Vet Clin North Am Equine Pract 36(1):87–103. 10.1016/j.cveq.2019.12.006 32145836 10.1016/j.cveq.2019.12.006

[16] Fan EKY, Fan J (2018) Regulation of alveolar macrophage death in acute lung inflammation. Respir Res 19(1):50. 10.1186/s12931-018-0756-5 29587748 10.1186/s12931-018-0756-5 PMC5872399

[17] Chen X, Liu C, Yu R, Gan Z, Zhang Z, Chen Z, Liu Y, Wu D, Yu X, Liu C, Cao Y (2023) Interaction between ferroptosis and TNF-alpha: impact in obesity-related osteoporosis. FASEB J 37(6):e22947. 10.1096/fj.202201958R 37199646 10.1096/fj.202201958R

[18] Wang W, Green M, Choi JE, Gijon M, Kennedy PD, Johnson JK, Liao P, Lang X, Kryczek I, Sell A, Xia H, Zhou J, Li G, Li J, Li W, Wei S, Vatan L, Zhang H, Szeliga W, Gu W, Liu R, Lawrence TS, Lamb C, Tanno Y, Cieslik M, Stone E, Georgiou G, Chan TA, Chinnaiyan A, Zou W (2019) CD8(+) T cells regulate tumour ferroptosis during cancer immunotherapy. Nature 569(7755):270–274. 10.1038/s41586-019-1170-y 31043744 10.1038/s41586-019-1170-y PMC6533917

[19] Zhu L (2010) Skp2 knockout reduces cell proliferation and mouse body size: and prevents cancer? Cell Res 20(6):605–607. 10.1038/cr.2010.71 20502441 10.1038/cr.2010.71 PMC2990532

[20] Yamauchi Y, Nita A, Nishiyama M, Muto Y, Shimizu H, Nakatsumi H, Nakayama KI (2020) Skp2 contributes to cell cycle progression in trophoblast stem cells and to placental development. Genes Cells 25(6):427–438. 10.1111/gtc.12769 32267063 10.1111/gtc.12769

[21] Miyake S, Murai S, Kakuta S, Uchiyama Y, Nakano H (2020) Identification of the hallmarks of necroptosis and ferroptosis by transmission electron microscopy. Biochem Biophys Res Commun 527(3):839–844. 10.1016/j.bbrc.2020.04.127 32430176 10.1016/j.bbrc.2020.04.127

[22] Soares-Silva M, Diniz FF, Gomes GN, Bahia D (2016) The mitogen-activated protein kinase (MAPK) pathway: role in immune evasion by trypanosomatids. Front Microbiol. 10.3389/fmicb.2016.00183 26941717 10.3389/fmicb.2016.00183 PMC4764696

[23] Lu Z, Xu S, Joazeiro C, Cobb MH, Hunter T (2002) The PHD domain of MEKK1 acts as an E3 ubiquitin ligase and mediates ubiquitination and degradation of ERK1/2. Mol Cell 9(5):945–956. 10.1016/s1097-2765(02)00519-1 12049732 10.1016/s1097-2765(02)00519-1

[24] Ndoja A, Reja R, Lee SH, Webster JD, Ngu H, Rose CM, Kirkpatrick DS, Modrusan Z, Chen YJ, Dugger DL, Gandham V, Xie L, Newton K, Dixit VM (2020) Ubiquitin ligase COP1 suppresses neuroinflammation by degrading c/EBPbeta in microglia. Cell 182(5):1156 e12-1169 e12. 10.1016/j.cell.2020.07.011 32795415 10.1016/j.cell.2020.07.011

[25] Jin H, Huang X, Pan Q, Ma N, Xie X, Wei Y, Yu F, Wen W, Zhang B, Zhang P, Chen X, Wang J, Liu RY, Lin J, Meng X, Lee MH (2024) The EIF3H-HAX1 axis increases RAF-MEK-ERK signaling activity to promote colorectal cancer progression. Nat Commun 15(1):2551. 10.1038/s41467-024-46521-3 38514606 10.1038/s41467-024-46521-3 PMC10957977

[26] Li X, Bian Y, Takizawa Y, Hashimoto T, Ikoma T, Tanaka J, Kitamura N, Inagaki Y, Komada M, Tanaka T (2013) ERK-dependent downregulation of Skp2 reduces Myc activity with HGF, leading to inhibition of cell proliferation through a decrease in Id1 expression. Mol Cancer Res 11(11):1437–1447. 10.1158/1541-7786.MCR-12-0718 24177224 10.1158/1541-7786.MCR-12-0718

[27] Chung YK, Chi-Hung Or R, Lu CH, Ouyang WT, Yang SY, Chang CC (2015) Sulforaphane down-regulates SKP2 to stabilize p27(KIP1) for inducing antiproliferation in human colon adenocarcinoma cells. J Biosci Bioeng 119(1):35–42. 10.1016/j.jbiosc.2014.06.009 25070589 10.1016/j.jbiosc.2014.06.009

[28] Kauko O, O’Connor CM, Kulesskiy E, Sangodkar J, Aakula A, Izadmehr S, Yetukuri L, Yadav B, Padzik A, Laajala TD, Haapaniemi P, Momeny M, Varila T, Ohlmeyer M, Aittokallio T, Wennerberg K, Narla G, Westermarck J (2018) PP2A inhibition is a druggable MEK inhibitor resistance mechanism in KRAS-mutant lung cancer cells. Sci Transl Med. 10.1126/scitranslmed.aaq1093 30021885 10.1126/scitranslmed.aaq1093 PMC8335581

[29] Petzold T, Zhang Z, Ballesteros I, Saleh I, Polzin A, Thienel M, Liu L, Ul Ain Q, Ehreiser V, Weber C, Kilani B, Mertsch P, Gotschke J, Cremer S, Fu W, Lorenz M, Ishikawa-Ankerhold H, Raatz E, El-Nemr S, Gorlach A, Marhuenda E, Stark K, Pircher J, Stegner D, Gieger C, Schmidt-Supprian M, Gaertner F, Almendros I, Kelm M, Schulz C, Hidalgo A, Massberg S (2022) Neutrophil “plucking” on megakaryocytes drives platelet production and boosts cardiovascular disease. Immunity 55(12):2285 e7-2299 e7. 10.1016/j.immuni.2022.10.001 36272416 10.1016/j.immuni.2022.10.001 PMC9767676

[30] Zhang J, Guo Y, Mak M, Tao Z (2024) Translational medicine for acute lung injury. J Transl Med 22(1):25. 10.1186/s12967-023-04828-7 38183140 10.1186/s12967-023-04828-7 PMC10768317

[31] Kulkarni HS, Lee JS, Bastarache JA, Kuebler WM, Downey GP, Albaiceta GM, Altemeier WA, Artigas A, Bates JHT, Calfee CS, Dela Cruz CS, Dickson RP, Englert JA, Everitt JI, Fessler MB, Gelman AE, Gowdy KM, Groshong SD, Herold S, Homer RJ, Horowitz JC, Hsia CCW, Kurahashi K, Laubach VE, Looney MR, Lucas R, Mangalmurti NS, Manicone AM, Martin TR, Matalon S, Matthay MA, McAuley DF, McGrath-Morrow SA, Mizgerd JP, Montgomery SA, Moore BB, Noel A, Perlman CE, Reilly JP, Schmidt EP, Skerrett SJ, Suber TL, Summers C, Suratt BT, Takata M, Tuder R, Uhlig S, Witzenrath M, Zemans RL, Matute-Bello G (2022) Update on the features and measurements of experimental acute lung injury in animals: an official american thoracic society workshop report. Am J Respir Cell Mol Biol 66(2):e1–e14. 10.1165/rcmb.2021-0531ST 35103557 10.1165/rcmb.2021-0531ST PMC8845128

[32] Loo DT (2011) In situ detection of apoptosis by the TUNEL assay: an overview of techniques. Methods Mol Biol 682:3–13. 10.1007/978-1-60327-409-8_1 21057916 10.1007/978-1-60327-409-8_1

[33] Liu J, Xia X, Huang P (2020) xCT: a critical molecule that links cancer metabolism to redox signaling. Mol Ther 28(11):2358–2366. 10.1016/j.ymthe.2020.08.021 32931751 10.1016/j.ymthe.2020.08.021 PMC7647670

[34] Zhou Q, Zhang J (2022) K27-linked noncanonic ubiquitination in immune regulation. J Leukoc Biol 111(1):223–235. 10.1002/JLB.4RU0620-397RR 33857334 10.1002/JLB.4RU0620-397RR

[35] Tracz M, Bialek W (2021) Beyond K48 and K63: non-canonical protein ubiquitination. Cell Mol Biol Lett 26(1):1. 10.1186/s11658-020-00245-6 33402098 10.1186/s11658-020-00245-6 PMC7786512

[36] Lee DH, Goldberg AL (1998) Proteasome inhibitors: valuable new tools for cell biologists. Trends Cell Biol 8(10):397–403. 10.1016/s0962-8924(98)01346-4 9789328 10.1016/s0962-8924(98)01346-4

[37] Ji CH, Kwon YT (2017) Crosstalk and interplay between the ubiquitin-proteasome system and autophagy. Mol Cells 40(7):441–449. 10.14348/molcells.2017.0115 28743182 10.14348/molcells.2017.0115 PMC5547213

[38] Yu X, Wang R, Zhang Y, Zhou L, Wang W, Liu H, Li W (2019) Skp2-mediated ubiquitination and mitochondrial localization of Akt drive tumor growth and chemoresistance to cisplatin. Oncogene 38(50):7457–7472. 10.1038/s41388-019-0955-7 31435020 10.1038/s41388-019-0955-7

[39] Wu T, Gu X, Cui H (2021) Emerging roles of SKP2 in cancer drug resistance. Cells. 10.3390/cells10051147 34068643 10.3390/cells10051147 PMC8150781

[40] Zhou L, Yu X, Li M, Gong G, Liu W, Li T, Zuo H, Li W, Gao F, Liu H (2020) Cdh1-mediated Skp2 degradation by dioscin reprogrammes aerobic glycolysis and inhibits colorectal cancer cells growth. EBioMedicine 51:102570. 10.1016/j.ebiom.2019.11.031 31806563 10.1016/j.ebiom.2019.11.031 PMC7000337

[41] Costa I, Barbosa DJ, Benfeito S, Silva V, Chavarria D, Borges F, Remiao F, Silva R (2023) Molecular mechanisms of ferroptosis and their involvement in brain diseases. Pharmacol Ther 244:108373. 10.1016/j.pharmthera.2023.108373 36894028 10.1016/j.pharmthera.2023.108373

[42] Macias-Rodriguez RU, Inzaugarat ME, Ruiz-Margain A, Nelson LJ, Trautwein C, Cubero FJ (2020) Reclassifying hepatic cell death during liver damage: ferroptosis—a novel form of non-apoptotic cell death? Int J Mol Sci. 10.3390/ijms21051651 32121273 10.3390/ijms21051651 PMC7084577

[43] He DH, Chen YF, Zhou YL, Zhang SB, Hong M, Yu X, Wei SF, Fan XZ, Li SY, Wang Q, Lu Y, Liu YQ (2021) Phytochemical library screening reveals betulinic acid as a novel Skp2-SCF E3 ligase inhibitor in non-small cell lung cancer. Cancer Sci 112(8):3218–3232. 10.1111/cas.15005 34080260 10.1111/cas.15005 PMC8353894

[44] Radmand A, Lokugamage MP, Kim H, Dobrowolski C, Zenhausern R, Loughrey D, Huayamares SG, Hatit MZC, Ni H, Del Cid A, Da Silva Sanchez AJ, Paunovska K, Schrader Echeverri E, Shajii A, Peck H, Santangelo PJ, Dahlman JE (2023) The transcriptional response to lung-targeting lipid nanoparticles in vivo. Nano Lett 23(3):993–1002. 10.1021/acs.nanolett.2c04479 36701517 10.1021/acs.nanolett.2c04479 PMC9912332

[45] Luo Y, Fan C, Yang M, Dong M, Bucala R, Pei Z, Zhang Y, Ren J (2020) CD74 knockout protects against LPS-induced myocardial contractile dysfunction through AMPK-Skp2-SUV39H1-mediated demethylation of BCLB. Br J Pharmacol 177(8):1881–1897. 10.1111/bph.14959 31877229 10.1111/bph.14959 PMC7070165

[46] Eisener-Dorman AF, Lawrence DA, Bolivar VJ (2009) Cautionary insights on knockout mouse studies: the gene or not the gene? Brain Behav Immun 23(3):318–324. 10.1016/j.bbi.2008.09.001 18822367 10.1016/j.bbi.2008.09.001 PMC2746382

[47] Li W, Zhang W, Deng M, Loughran P, Tang Y, Liao H, Zhang X, Liu J, Billiar TR, Lu B (2018) Stearoyl lysophosphatidylcholine inhibits endotoxin-induced caspase-11 activation. Shock 50(3):339–345. 10.1097/SHK.0000000000001012 28991049 10.1097/SHK.0000000000001012 PMC5882614

[48] Dimitrov-Markov S, Perales-Paton J, Bockorny B, Dopazo A, Munoz M, Banos N, Bonilla V, Menendez C, Duran Y, Huang L, Perea S, Muthuswamy SK, Al-Shahrour F, Lopez-Casas PP, Hidalgo M (2020) Discovery of new targets to control metastasis in pancreatic cancer by single-cell transcriptomics analysis of circulating tumor cells. Mol Cancer Ther 19(8):1751–1760. 10.1158/1535-7163.MCT-19-1166 32499301 10.1158/1535-7163.MCT-19-1166

[49] Chen Y-S, Chuang W-C, Kung H-N, Cheng C-Y, Huang D-Y, Sekar P, Lin W-W (2022) Pan-caspase inhibitor zVAD induces necroptotic and autophagic cell death in TLR3/4-stimulated macrophages. Mol Cells 45(4):257–272. 10.14348/molcells.2021.0193 34949739 10.14348/molcells.2021.0193 PMC9001149

[50] Cao L, Mu W (2021) Necrostatin-1 and necroptosis inhibition: pathophysiology and therapeutic implications. Pharmacol Res 163:105297. 10.1016/j.phrs.2020.105297 33181319 10.1016/j.phrs.2020.105297 PMC7962892

[51] Ding Z, Liang X, Wang J, Song Z, Guo Q, Schafer MKE, Huang C (2023) Inhibition of spinal ferroptosis-like cell death alleviates hyperalgesia and spontaneous pain in a mouse model of bone cancer pain. Redox Biol 62:102700. 10.1016/j.redox.2023.102700 37084690 10.1016/j.redox.2023.102700 PMC10141498

[52] Qu M, Wang Y, Qiu Z, Zhu S, Guo K, Chen W, Miao C, Zhang H (2022) Necroptosis, pyroptosis, ferroptosis in sepsis and treatment. Shock 57(6):161–171. 10.1097/SHK.0000000000001936 35759299 10.1097/SHK.0000000000001936

[53] Chen H, Li Y, Wu J, Li G, Tao X, Lai K, Yuan Y, Zhang X, Zou Z, Xu Y (2020) RIPK3 collaborates with GSDMD to drive tissue injury in lethal polymicrobial sepsis. Cell Death Differ 27(9):2568–2585. 10.1038/s41418-020-0524-1 32152555 10.1038/s41418-020-0524-1 PMC7429874

[54] Li N, Zhou H, Wu H, Wu Q, Duan M, Deng W, Tang Q (2019) STING-IRF3 contributes to lipopolysaccharide-induced cardiac dysfunction, inflammation, apoptosis and pyroptosis by activating NLRP3. Redox Biol. 10.1016/j.redox.2019.101215 31121492 10.1016/j.redox.2019.101215 PMC6529775

[55] Chang F, Steelman LS, Lee JT, Shelton JG, Navolanic PM, Blalock WL, Franklin RA, McCubrey JA (2003) Signal transduction mediated by the Ras/Raf/MEK/ERK pathway from cytokine receptors to transcription factors: potential targeting for therapeutic intervention. Leukemia 17(7):1263–1293. 10.1038/sj.leu.2402945 12835716 10.1038/sj.leu.2402945

[56] Yagoda N, von Rechenberg M, Zaganjor E, Bauer AJ, Yang WS, Fridman DJ, Wolpaw AJ, Smukste I, Peltier JM, Boniface JJ, Smith R, Lessnick SL, Sahasrabudhe S, Stockwell BR (2007) RAS–RAF–MEK-dependent oxidative cell death involving voltage-dependent anion channels. Nature 447(7146):865–869. 10.1038/nature05859 10.1038/nature05859 PMC3047570 17568748

[57] Lucas RM, Luo L, Stow JL (2022) ERK1/2 in immune signalling. Biochem Soc Trans 50(5):1341–1352. 10.1042/BST20220271 36281999 10.1042/BST20220271 PMC9704528

[58] Demirdizen E, Al-Ali R, Narayanan A, Sun X, Varga JP, Steffl B, Brom M, Krunic D, Schmidt C, Schmidt G, Bestvater F, Taranda J, Turcan S (2023) TRIM67 drives tumorigenesis in oligodendrogliomas through Rho GTPase-dependent membrane blebbing. Neuro Oncol 25(6):1031–1043. 10.1093/neuonc/noac233 36215168 10.1093/neuonc/noac233 PMC10237422

[59] Liu MR, Zhu WT, Pei DS (2021) System Xc(-): a key regulatory target of ferroptosis in cancer. Investig New Drugs 39(4):1123–1131. 10.1007/s10637-021-01070-0 33506324 10.1007/s10637-021-01070-0

[60] Sorada T, Morimoto D, Walinda E, Sugase K (2021) Molecular recognition and deubiquitination of cyclic K48-linked ubiquitin chains by OTUB1. Biochem Biophys Res Commun 562:94–99. 10.1016/j.bbrc.2021.05.031 34049206 10.1016/j.bbrc.2021.05.031

[61] Swatek KN, Komander D (2016) Ubiquitin modifications. Cell Res 26(4):399–422. 10.1038/cr.2016.39 27012465 10.1038/cr.2016.39 PMC4822133

[62] Senft D, Qi J, Ronai ZA (2018) Ubiquitin ligases in oncogenic transformation and cancer therapy. Nat Rev Cancer 18(2):69–88. 10.1038/nrc.2017.105 29242641 10.1038/nrc.2017.105 PMC6054770

[63] Behrends C, Harper JW (2011) Constructing and decoding unconventional ubiquitin chains. Nat Struct Mol Biol 18(5):520–528. 10.1038/nsmb.2066 21540891 10.1038/nsmb.2066

[64] Britt EC, Lika J, Giese MA, Schoen TJ, Seim GL, Huang Z, Lee PY, Huttenlocher A, Fan J (2022) Switching to the cyclic pentose phosphate pathway powers the oxidative burst in activated neutrophils. Nat Metab 4(3):389–403. 10.1038/s42255-022-00550-8 35347316 10.1038/s42255-022-00550-8 PMC8964420

[65] Kolla S, Ye M, Mark KG, Rape M (2022) Assembly and function of branched ubiquitin chains. Trends Biochem Sci 47(9):759–771. 10.1016/j.tibs.2022.04.003 35508449 10.1016/j.tibs.2022.04.003

[66] Yau RG, Doerner K, Castellanos ER, Haakonsen DL, Werner A, Wang N, Yang XW, Martinez-Martin N, Matsumoto ML, Dixit VM, Rape M (2017) Assembly and function of heterotypic ubiquitin chains in cell-cycle and protein quality control. Cell 171(4):918 e20-933 e20. 10.1016/j.cell.2017.09.040 29033132 10.1016/j.cell.2017.09.040 PMC5669814

[67] Kaiho-Soma A, Akizuki Y, Igarashi K, Endo A, Shoda T, Kawase Y, Demizu Y, Naito M, Saeki Y, Tanaka K, Ohtake F (2021) TRIP12 promotes small-molecule-induced degradation through K29/K48-branched ubiquitin chains. Mol Cell 81(7):1411 e7-1424 e7. 10.1016/j.molcel.2021.01.023 33567268 10.1016/j.molcel.2021.01.023

[68] Pluska L, Jarosch E, Zauber H, Kniss A, Waltho A, Bagola K, von Delbruck M, Lohr F, Schulman BA, Selbach M, Dotsch V, Sommer T (2021) The UBA domain of conjugating enzyme Ubc1/Ube2K facilitates assembly of K48/K63-branched ubiquitin chains. EMBO J 40(6):e106094. 10.15252/embj.2020106094 33576509 10.15252/embj.2020106094 PMC7957398

[69] Ohtake F, Saeki Y, Ishido S, Kanno J, Tanaka K (2016) The K48–K63 branched ubiquitin chain regulates NF-kappaB signaling. Mol Cell 64(2):251–266. 10.1016/j.molcel.2016.09.014 27746020 10.1016/j.molcel.2016.09.014

[70] Beck DB, Werner A, Kastner DL, Aksentijevich I (2022) Disorders of ubiquitylation: unchained inflammation. Nat Rev Rheumatol 18(8):435–447. 10.1038/s41584-022-00778-4 35523963 10.1038/s41584-022-00778-4 PMC9075716

[71] Lv S, Han M, Yi R, Kwon S, Dai C, Wang R (2014) Anti-TNF-alpha therapy for patients with sepsis: a systematic meta-analysis. Int J Clin Pract 68(4):520–528. 10.1111/ijcp.12382 24548627 10.1111/ijcp.12382

[72] Chen W, Liu J, Ge F, Chen Z, Qu M, Nan K, Gu J, Jiang Y, Gao S, Liao Y, Wang C, Zhang H, Miao C (2022) Long noncoding RNA HOTAIRM1 promotes immunosuppression in sepsis by inducing T cell exhaustion. J Immunol 208(3):618–632. 10.4049/jimmunol.2100709 35022270 10.4049/jimmunol.2100709

[73] Truong B, Allegri G, Liu XB, Burke KE, Zhu X, Cederbaum SD, Haberle J, Martini PGV, Lipshutz GS (2019) Lipid nanoparticle-targeted mRNA therapy as a treatment for the inherited metabolic liver disorder arginase deficiency. Proc Natl Acad Sci USA 116(42):21150–21159. 10.1073/pnas.1906182116 31501335 10.1073/pnas.1906182116 PMC6800360

[74] Vogel AB, Kanevsky I, Che Y, Swanson KA, Muik A, Vormehr M, Kranz LM, Walzer KC, Hein S, Guler A, Loschko J, Maddur MS, Ota-Setlik A, Tompkins K, Cole J, Lui BG, Ziegenhals T, Plaschke A, Eisel D, Dany SC, Fesser S, Erbar S, Bates F, Schneider D, Jesionek B, Sanger B, Wallisch AK, Feuchter Y, Junginger H, Krumm SA, Heinen AP, Adams-Quack P, Schlereth J, Schille S, Kroner C, de la Caridad Guimil Garcia R, Hiller T, Fischer L, Sellers RS, Choudhary S, Gonzalez O, Vascotto F, Gutman MR, Fontenot JA, Hall-Ursone S, Brasky K, Griffor MC, Han S, Su AAH, Lees JA, Nedoma NL, Mashalidis EH, Sahasrabudhe PV, Tan CY, Pavliakova D, Singh G, Fontes-Garfias C, Pride M, Scully IL, Ciolino T, Obregon J, Gazi M, Carrion R Jr, Alfson KJ, Kalina WV, Kaushal D, Shi PY, Klamp T, Rosenbaum C, Kuhn AN, Tureci O, Dormitzer PR, Jansen KU, Sahin U (2021) BNT162b vaccines protect rhesus macaques from SARS-CoV-2. Nature 592(7853):283–289. 10.1038/s41586-021-03275-y 33524990 10.1038/s41586-021-03275-y

[75] Rohner E, Yang R, Foo KS, Goedel A, Chien KR (2022) Unlocking the promise of mRNA therapeutics. Nat Biotechnol 40(11):1586–1600. 10.1038/s41587-022-01491-z 36329321 10.1038/s41587-022-01491-z

[76] Robinson E, MacDonald KD, Slaughter K, McKinney M, Patel S, Sun C, Sahay G (2018) Lipid nanoparticle-delivered chemically modified mRNA restores chloride secretion in cystic fibrosis. Mol Ther 26(8):2034–2046. 10.1016/j.ymthe.2018.05.014 29910178 10.1016/j.ymthe.2018.05.014 PMC6094356

[77] Singer M, Deutschman CS, Seymour CW, Shankar-Hari M, Annane D, Bauer M, Bellomo R, Bernard GR, Chiche JD, Coopersmith CM, Hotchkiss RS, Levy MM, Marshall JC, Martin GS, Opal SM, Rubenfeld GD, van der Poll T, Vincent JL, Angus DC (2016) The third international consensus definitions for sepsis and septic shock (sepsis-3). JAMA 315(8):801–810. 10.1001/jama.2016.0287 26903338 10.1001/jama.2016.0287 PMC4968574

[78] Force ADT, Ranieri VM, Rubenfeld GD, Thompson BT, Ferguson ND, Caldwell E, Fan E, Camporota L, Slutsky AS (2012) Acute respiratory distress syndrome: the Berlin definition. JAMA 307(23):2526–2533. 10.1001/jama.2012.5669 22797452 10.1001/jama.2012.5669

[79] Chen H, Zhang H, Wang K, Yao Y, Xiao X (2019) Minimum quality threshold in pre-clinical sepsis studies (MQTiPSS): an international expert consensus initiative for improvement of animal modeling in sepsis. Zhonghua Wei Zhong Bing Ji Jiu Yi Xue 31(8):930–932. 10.3760/cma.j.issn.2095-4352.2019.08.003 31537213 10.3760/cma.j.issn.2095-4352.2019.08.003

