# Supplementary material for: Suppression of Skp2 contributes to sepsis-induced acute lung injury by enhancing ferroptosis through the ubiquitination of SLC3A2
Source: Cell Mol Life Sci. 2024 Jul 30;81(1):325. doi: 10.1007/s00018-024-05348-3 (PMC11335248; doi:10.1007/s00018-024-05348-3)

Supplemental Figure 1

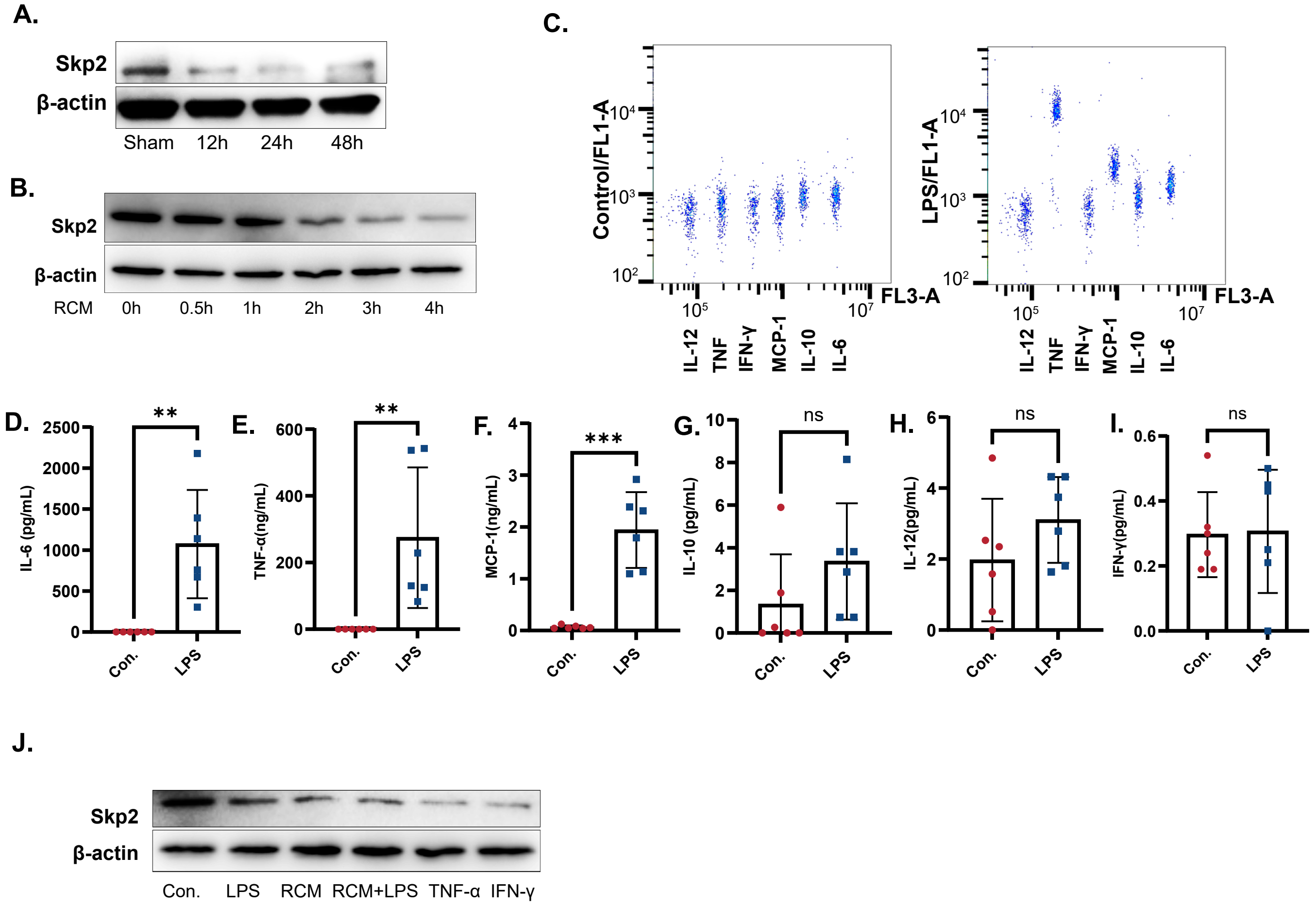

Supplemental Figure 2

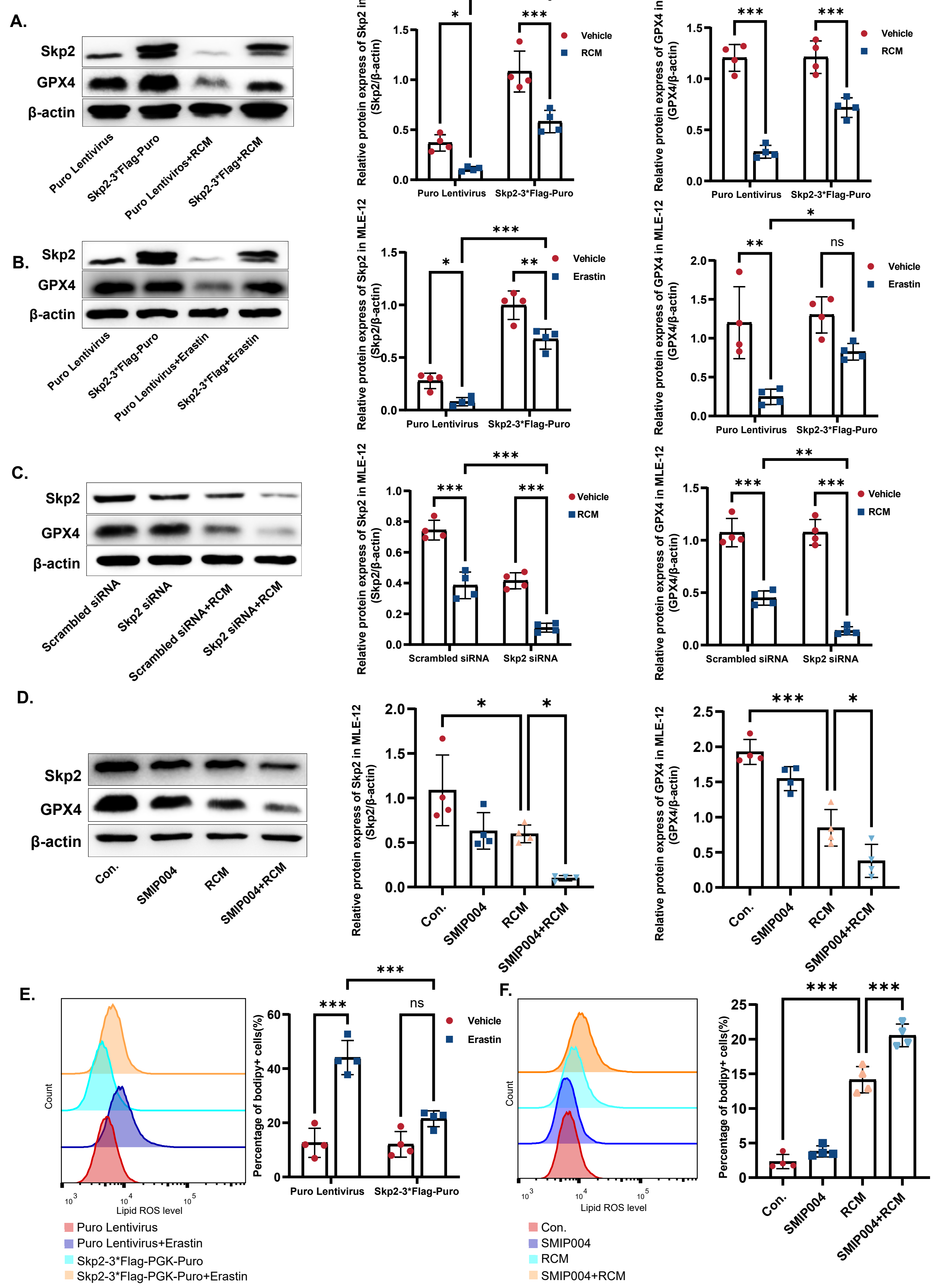

Supplemental Figure 3

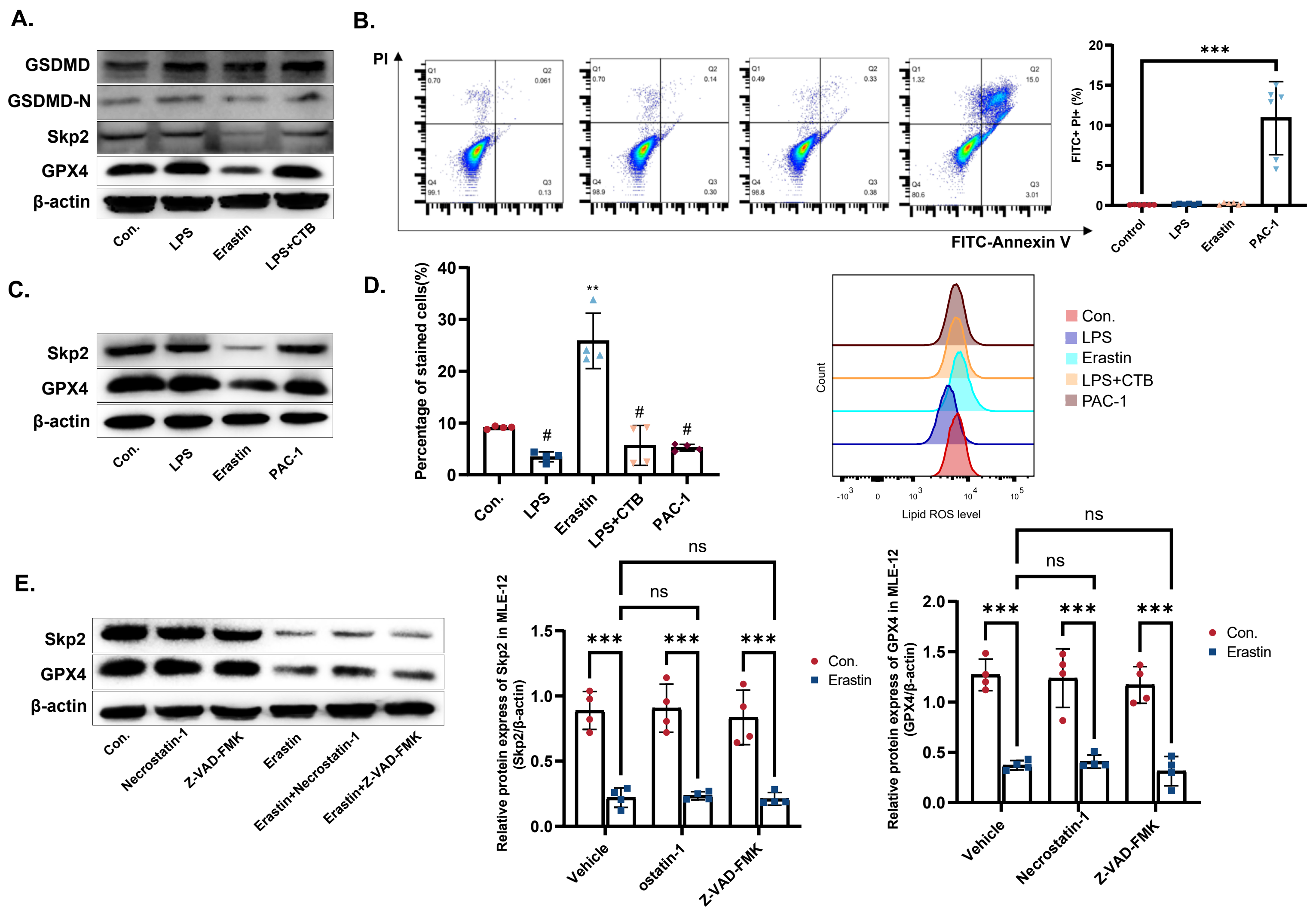

Supplemental Figure 4

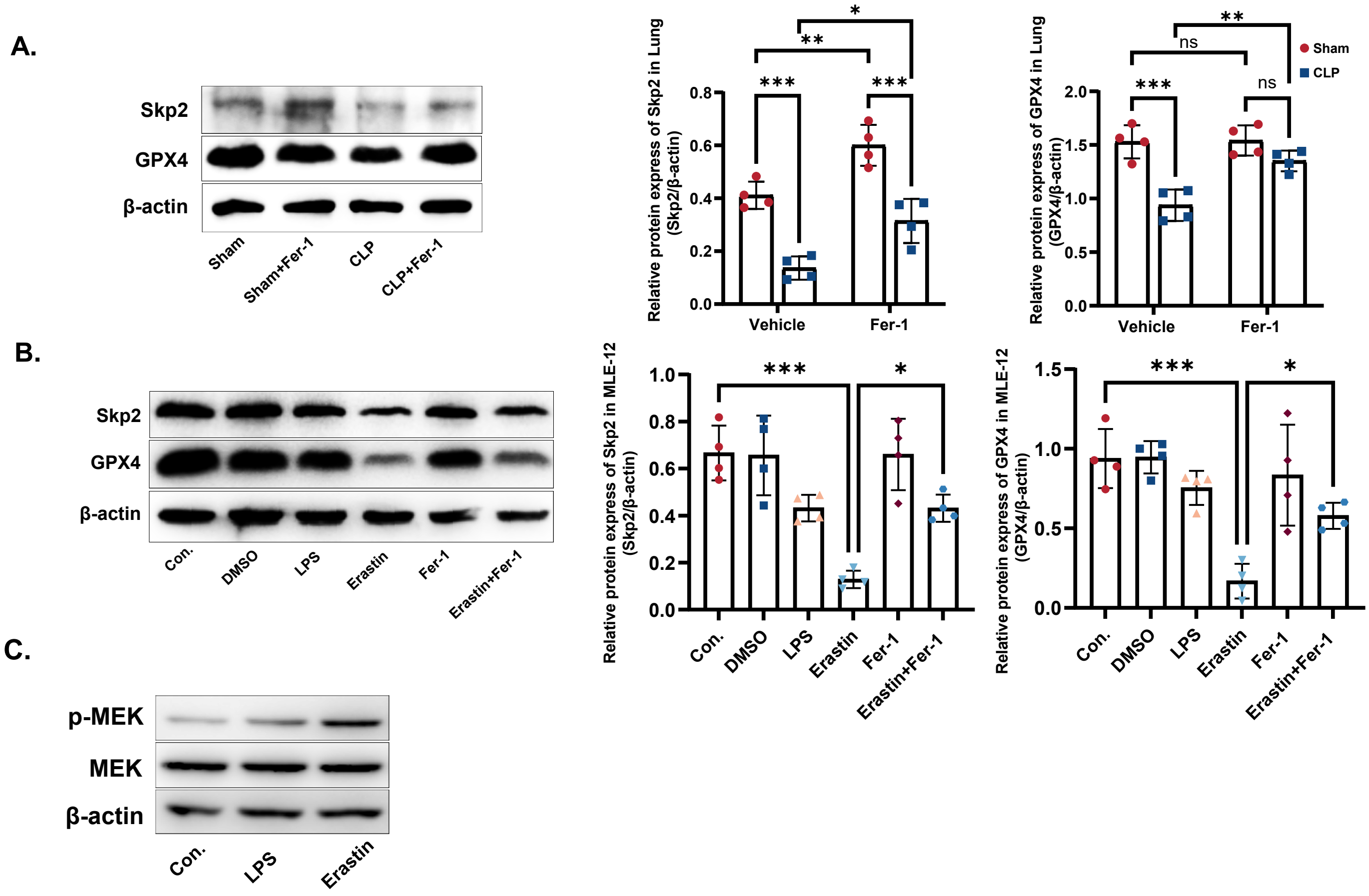

Supplemental Figure 5

A.

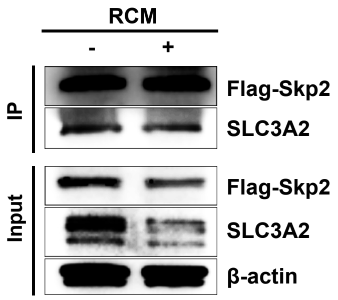

Supplemental Figure 6

A.

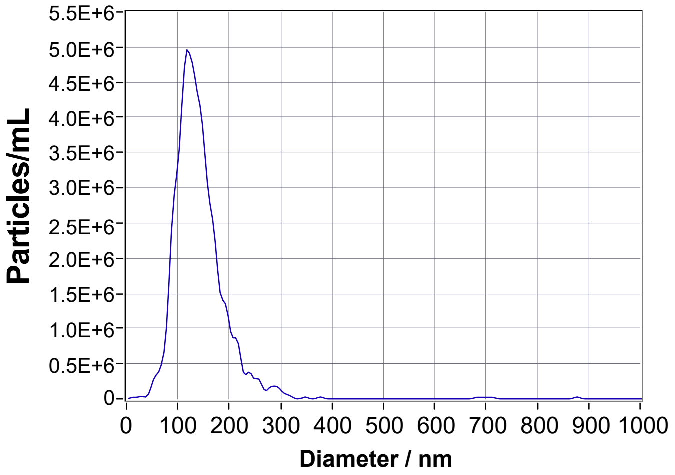

B.

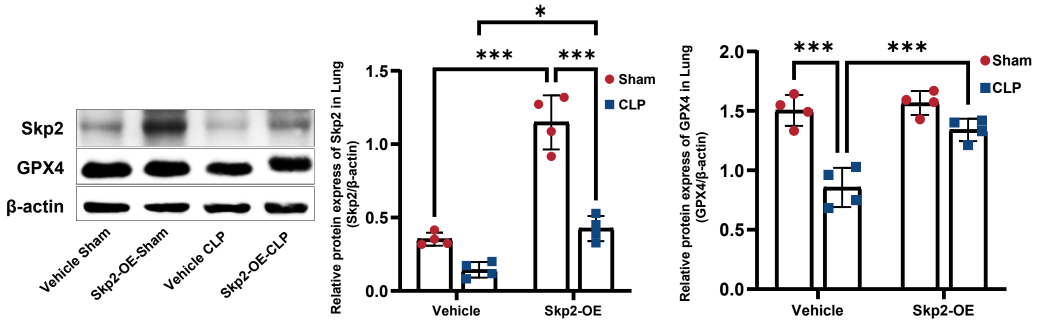

Supplement: Supplementary file 1 — Supplementary file1 Figure S1. The inflammatory cytokine storm inhibited Skp2 expression. (A) Skp2 protein expression in murine lungs at 12, 24 and 48 hours after CLP was measured by Western blotting; (B) MLE-12 cells were stimulated with RCM for varying time intervals (0.5, 1, 2, 3, and 4 hours), and Skp2 protein expression levels were measured by Western blotting; (C) The concentrations of cytokines in RCM were quantified using the Cytometric Bead Array (CBA). (D-I) Statistical analysis was conducted to determine the concentrations of IL-6, TNF-α, MCP-1, IL-10, IL-12, and IFN-γ in the supernatants of Raw264.7 cells treated with control or LPS (RCM). (J) MLE-12 cells were stimulated with 300 ng/mL TNF-α and 10 ng/mL IFN-γ, and Skp2 protein levels in each group were measured by western blotting. The data are presented as the means ± standard deviations (ns p>0.05, *P<0.05, **P<0.01, ***P<0.001). Figure S2. The inhibition of Skp2 exacerbated ferroptosis in the lung epithelium. (A-B) MLE-12 lung epithelial cells were transfected with Skp2 lentivirus (Skp2-3*Flag-PGK-Puro) or control lentivirus and selected with puromycin. The protein expression levels of Skp2 and GPX4 were measured by western blotting after lentivirus transfection and treatment with RCM(A) or Erastin (B). (C-D) Skp2 in MLE-12 cells was knocked down by siRNA(C) or inhibited by Skp2 inhibitor SMIP004(D), and the cells were treated with RCM. The protein expression levels of Skp2 and GPX4 in MLE-12 cells were measured by western blotting. Lipid peroxidation was analyzed by Bodipy fluorescence by flow cytometry after Erastin (E) or SMIP004 (F) treatment. The data are presented as the means ± standard deviations (ns P>0.05, *P<0.05, **P<0.01, ***P<0.001). Figure S3. Skp2 inhibition mainly occurs during ferroptosis. (A) Pyroptosis of MLE-12 cells was induced by LPS+CTB, and the expression of Skp2, GPX4 as and the N-terminal protein GSDMD was measured by western blotting. (B-C) The procaspase-3 activator PAC- [file 18_2024_5348_MOESM1_ESM.pdf]
