# Supplementary material for: Suppression of Skp2 contributes to sepsis-induced acute lung injury by enhancing ferroptosis through the ubiquitination of SLC3A2
Source: Cell Mol Life Sci. 2024 Jul 30;81(1):325. doi: 10.1007/s00018-024-05348-3 (PMC11335248; doi:10.1007/s00018-024-05348-3)
Supplement: Supplementary file 2 — Supplementary file1 (PDF 1562 kb) [file 18_2024_5348_MOESM2_ESM.pdf]

Figure 1C

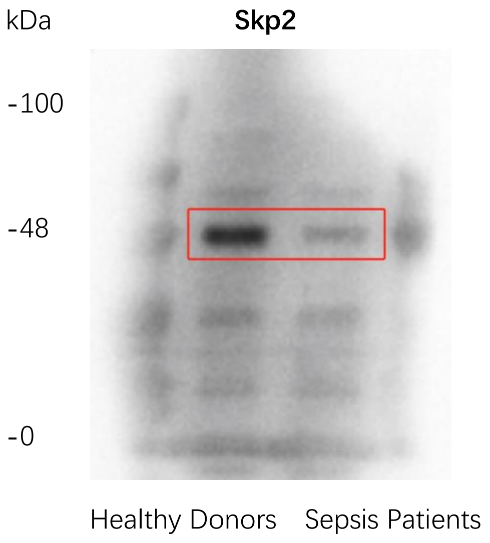

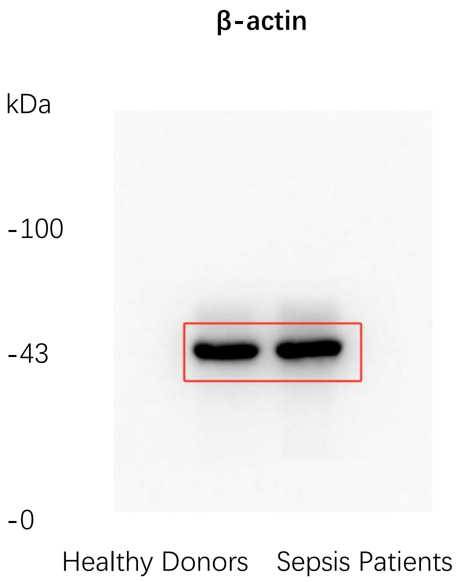

Figure 1D

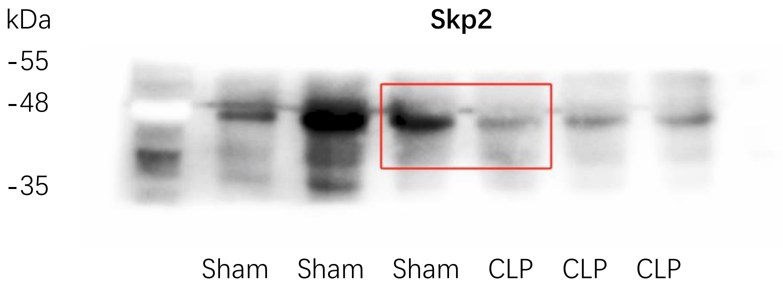

β-actin

kDa
-55
-43
-35

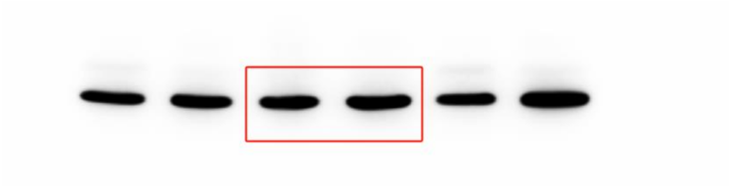

Sham Sham Sham CLP CLP CLP

Figure 1G

Skp2

kDa
-70
-48
-35

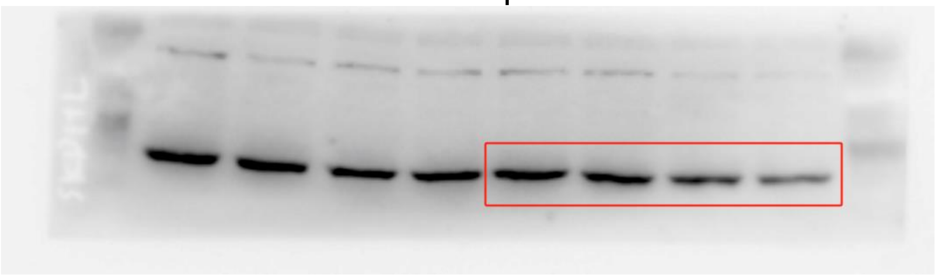

Con. LPS RCM RCM+LPS Con. LPS RCM RCM+LPS

β-actin

kDa
-70
-43
-35

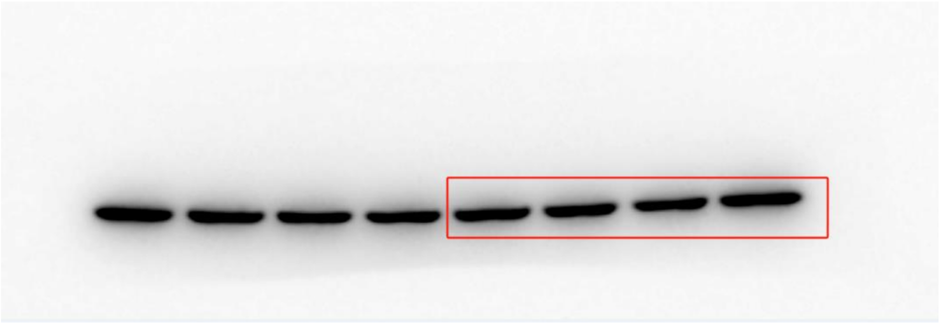

Con. LPS RCM RCM+LPS Con. LPS RCM RCM+LPS

Figure 1J

Skp2

kDa
-70
-48
-35

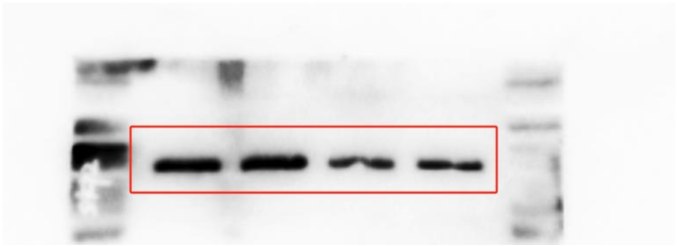

Con. LPS MCM MCM+LPS

β-actin

kDa

-70

-43

-35

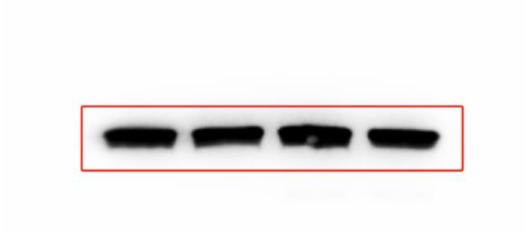

Con. LPS MCM MCM+LPS

Figure 2E

Skp2

kDa

-100

-48

-00

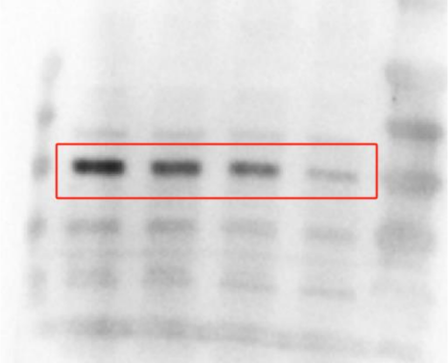

WT	+	-	+	-
Skp2$^{+/-}$	-	+	-	+
CLP	-	-	+	+

GPX4

kDa

-100

-20

-0

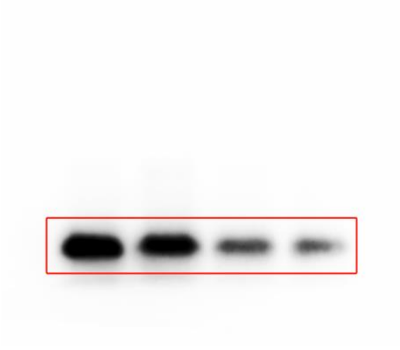

WT	+	-	+	-
Skp2$^{+/-}$	-	+	-	+
CLP	-	-	+	+

β-actin

kDa

-100

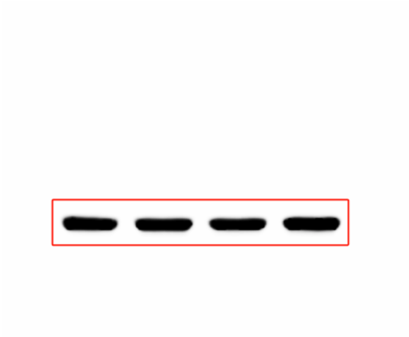

-43

-0

WT	+	-	+	-
Skp2$^{+/-}$	-	+	-	+
CLP	-	-	+	+

Figure 3A

Skp2

kDa

-200

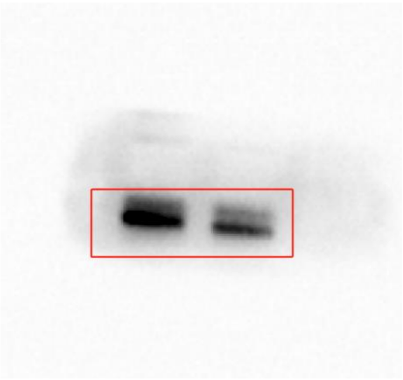

-48

-0

Con. RCM

p-MEK

kDa

-200

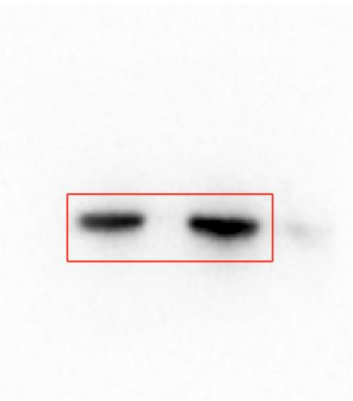

-45

-0

Con. RCM

MEK

kDa
-200
-45
-0

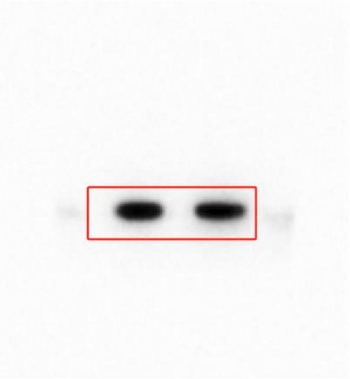

Con. RCM

p-P38

kDa
-200
-43
-0

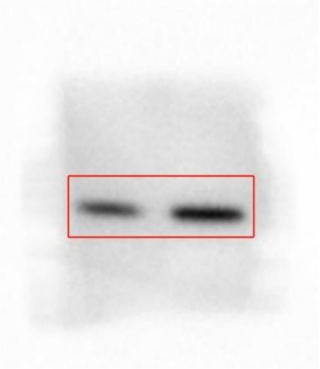

Con. RCM

P38

kDa
-200
-40
-0

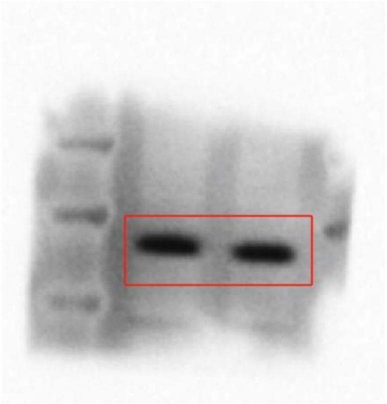

Con. RCM

p-JNK

kDa
-200
-54
-46
-0

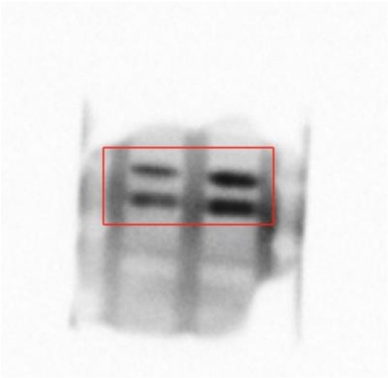

Con.   RCM

JNK

kDa
-200
-54
-46
-0

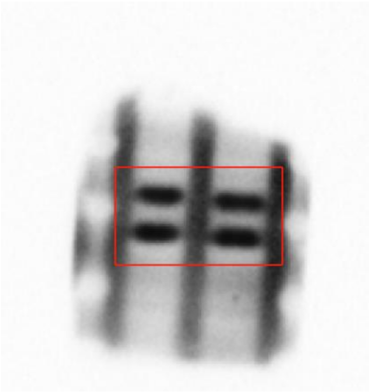

Con.   RCM

β-actin

kDa
-200
-43
-0

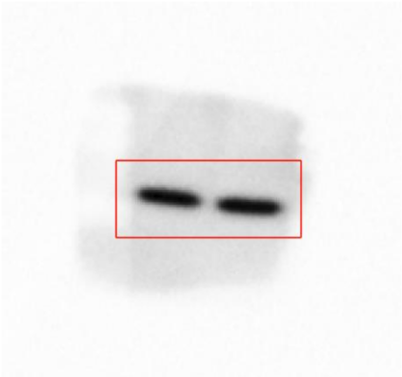

Con.   RCM

Figure 3B

Skp2

kDa
-200
-48
-0

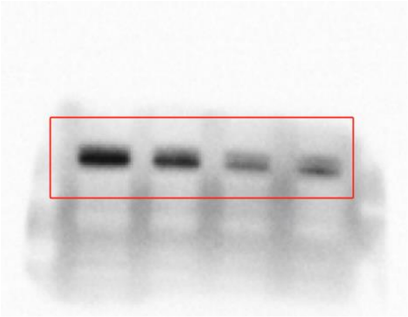

RCM - + + +
Con. iMEK iP38 iJNK

p-MEK

kDa
-200
-45
-0

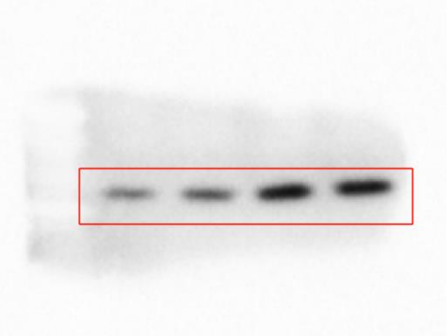

RCM - + + +
Con. iMEK iP38 iJNK

MEK

kDa
-200
-45
-0

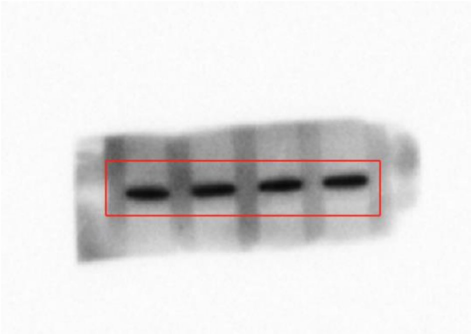

RCM - + + +
Con. iMEK iP38 iJNK

p-P38

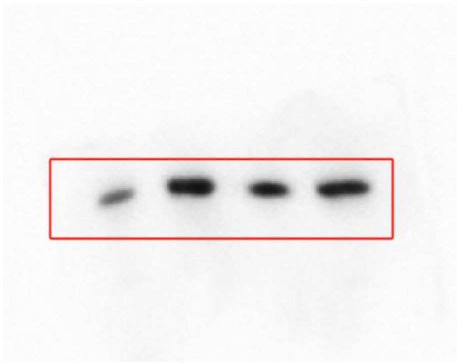

P38

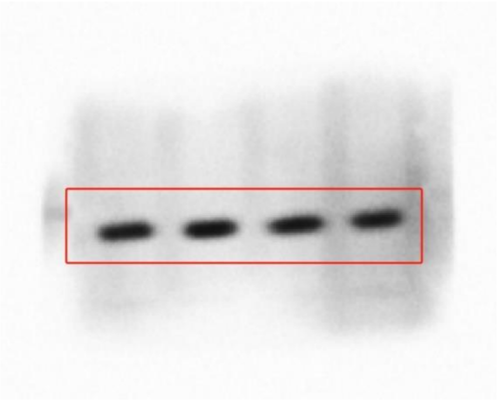

p-JNK

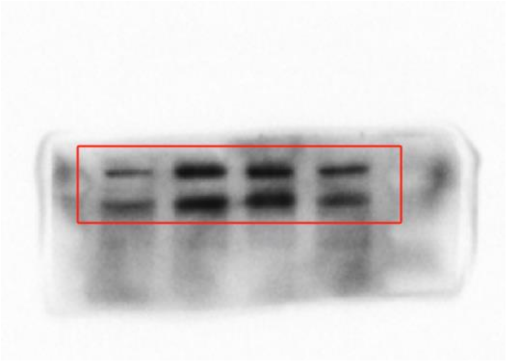

JNK

kDa

-200

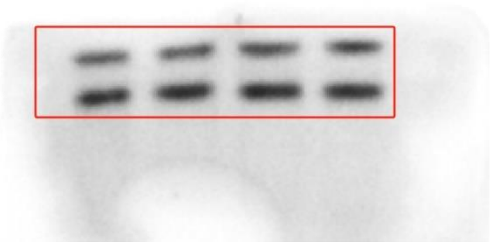

-54

-46

-0

RCM - + + +

Con. iMEK iP38 iJNK

β-actin

kDa

-200

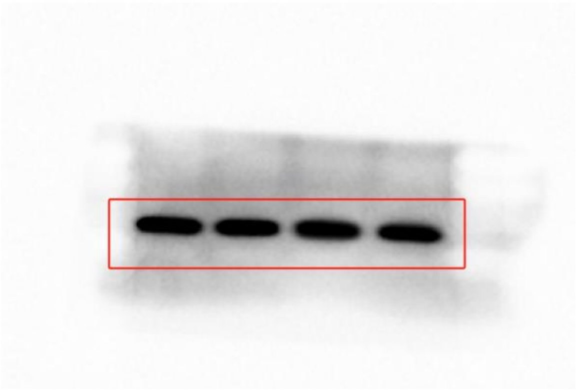

-43

-0

RCM - + + +

Con. iMEK iP38 iJNK

Figure 3C

Skp2

kDa

-200

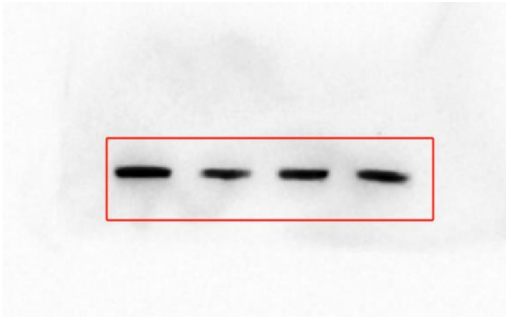

-48

-0

Con. RCM aMEK aERK

p-MEK

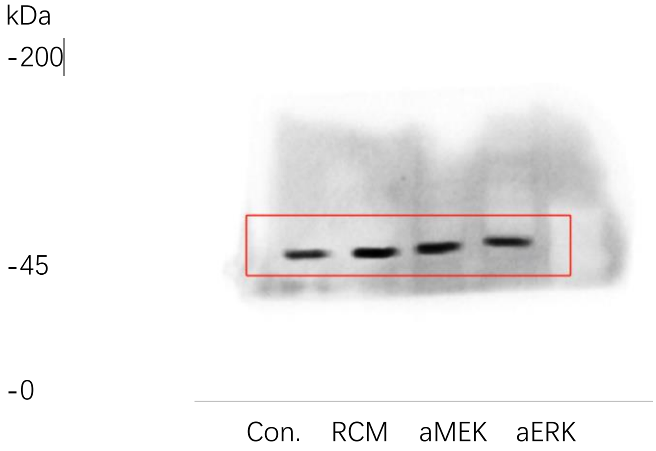

MEK

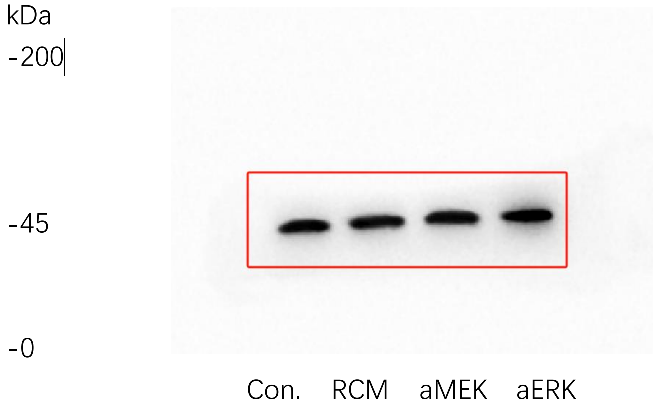

p-ERK

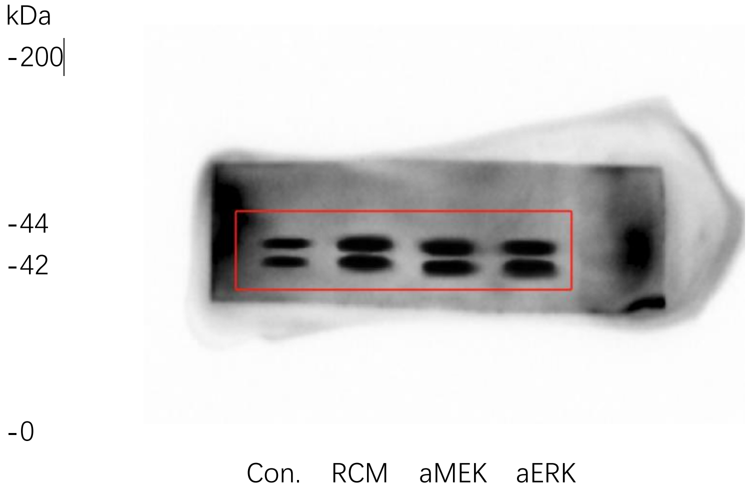

ERK

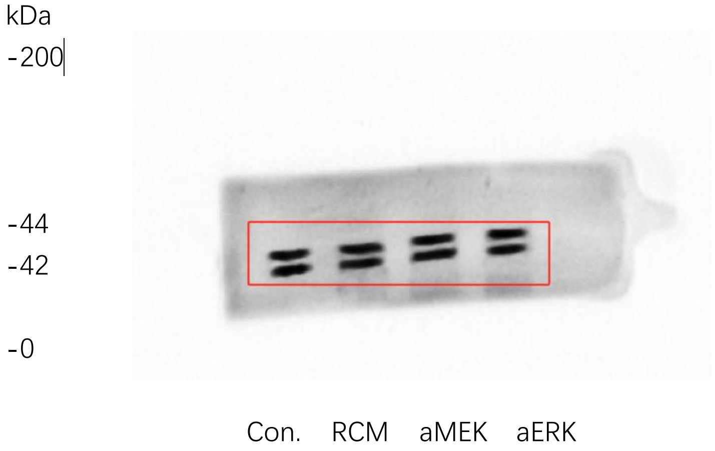

β-actin

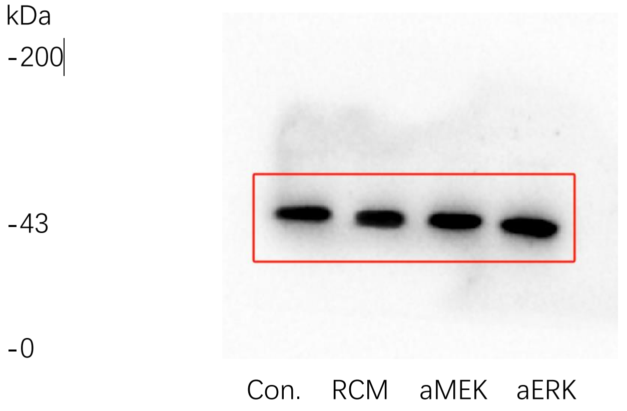

Figure 3D

Skp2

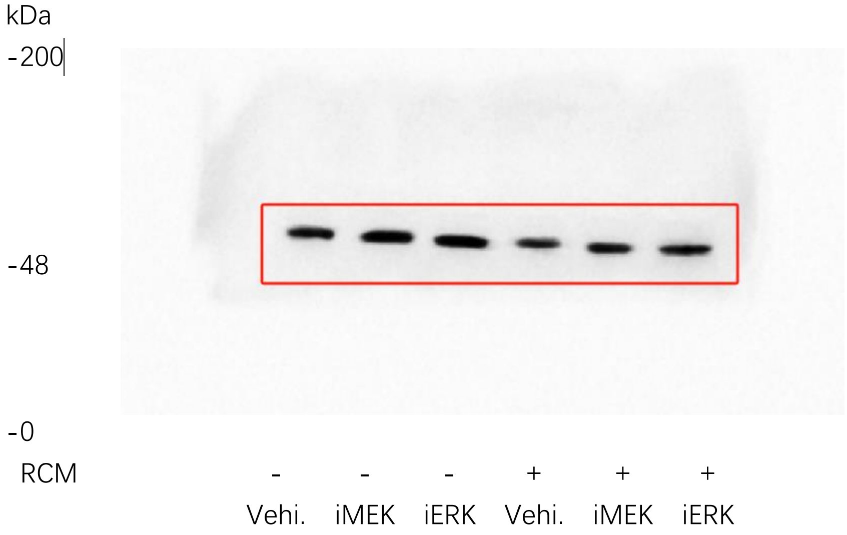

β-actin

kDa
-200

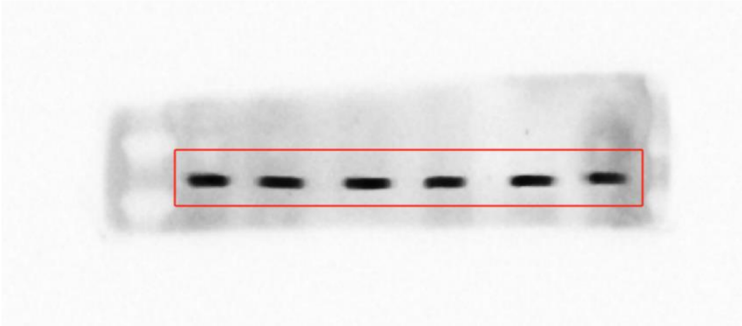

-43
-0

RCM - - - + + +

Vehi. iMEK iERK Vehi. iMEK iERK

Figure 3E

Skp2

kDa
-70
-48

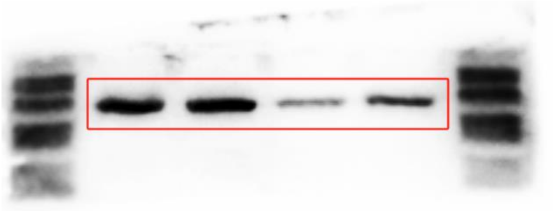

-35

Con. iMEK RCM iMEK+RCM

GPX4

kDa
-35
-20

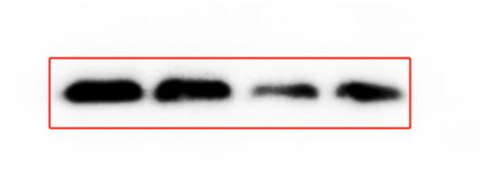

-0

Con. iMEK RCM iMEK+RCM

β-actin

kDa
-70
-43

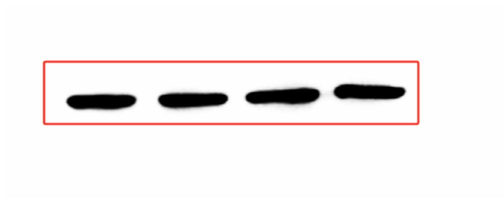

-35

Con. iMEK RCM iMEK+RCM

Figure 5B

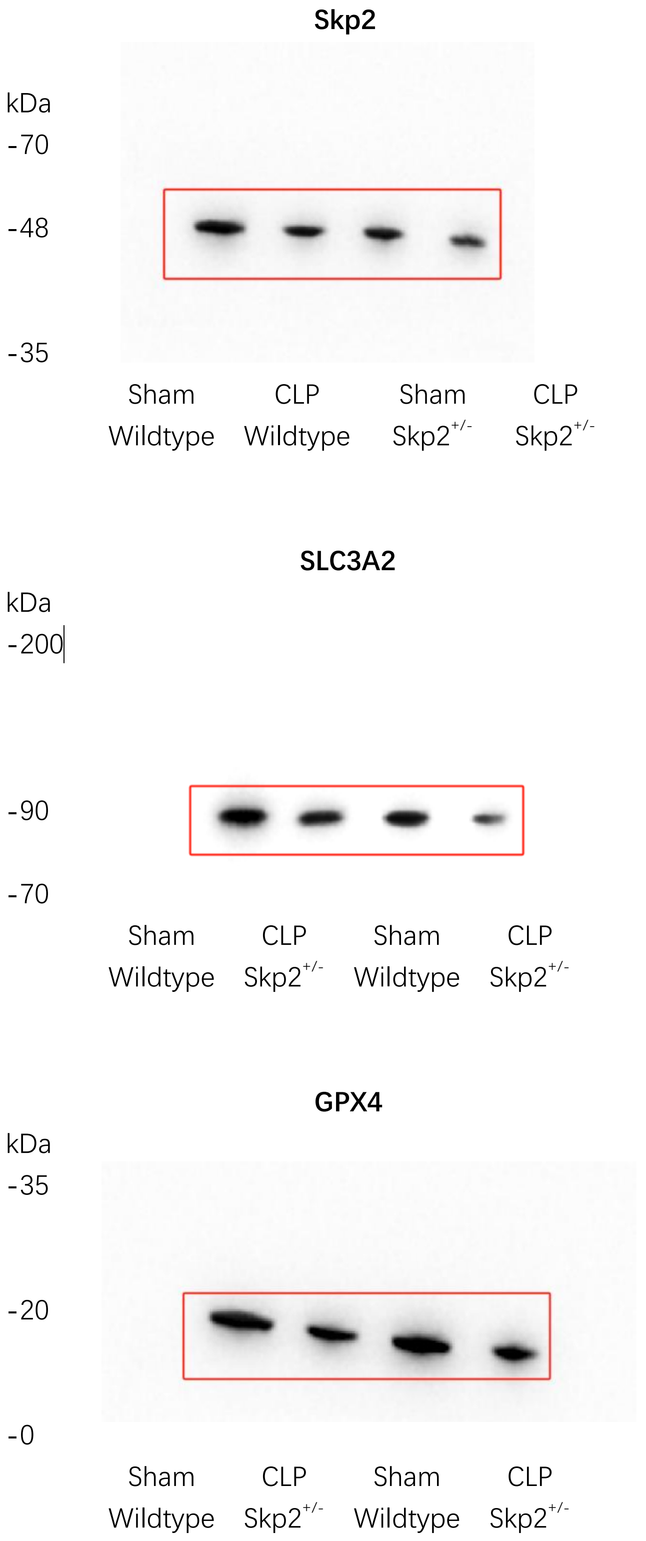
Skp2
kDa
-70
-48
-35
Sham Wildtype
CLP Wildtype
Sham Skp2+/-
CLP Skp2+/-
SLC3A2
kDa
-200
-90
-70
Sham Wildtype
CLP Skp2+/-
Sham Wildtype
CLP Skp2+/-
GPX4
kDa
-35
-20
-0
Sham Wildtype
CLP Skp2+/-
Sham Wildtype
CLP Skp2+/-

β-actin

kDa
-70
-43
-0

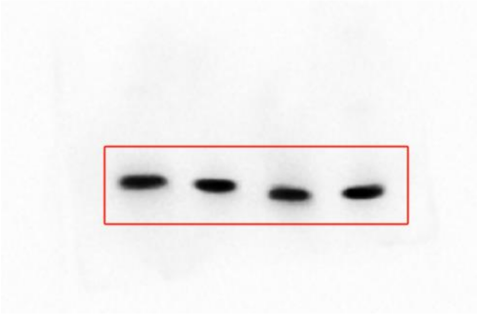

Sham	CLP	Sham	CLP
Wildtype	Skp2$^{+/-}$	Wildtype	Skp2$^{+/-}$

Figure 4C

Input-Flag

kDa
-70
-48
-25

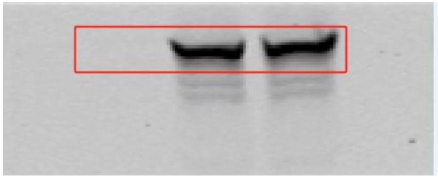

Flag-Skp2	-	+	+
Myc-SLC3A2	-	-	+

Input-Myc

kDa
-130
-90
-70

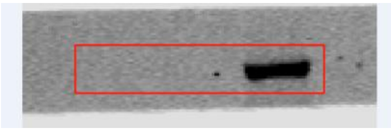

Flag-Skp2	-	+	+
Myc-SLC3A2	-	-	+

Input-GAPDH

kDa
-100
-36
-0

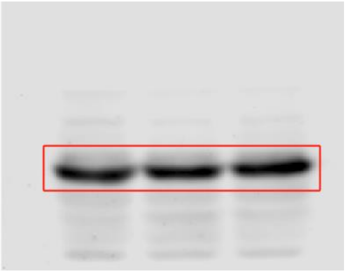

Flag-Skp2	-	+	+
Myc-SLC3A2	-	-	+

IP-Ub

kDa

-200

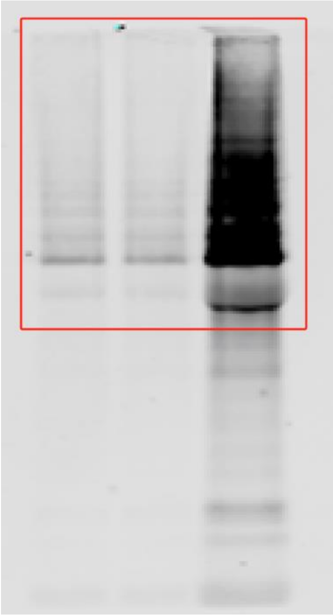

-0

Flag-Skp2	-	+	+
Myc-SLC3A2	-	-	+

Figure 4D

Myc-SLC3A2

Flag-Skp2

HA-Ub-WT | **HA-Ub-WT+Flag-Skp2** | **Empty** | **HA-Ub-WT** | **HA-Ub-WT** | **HA-Ub-K6R** | **HA-Ub-K11R** | **HA-Ub-K27R** | **HA-Ub-K29R** | **HA-Ub-K33R** | **HA-Ub-K48R** | **HA-Ub-K63R**

IP-Ub

kDa

-200

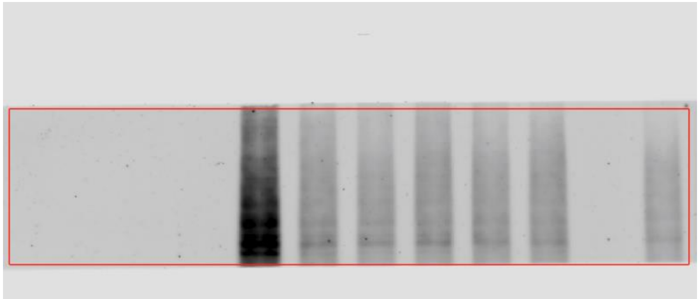

-=0

Input-Flag

kDa
-70
-48
-25

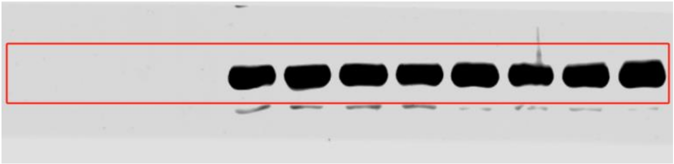

Input-Myc

kDa
-170
-90
-70

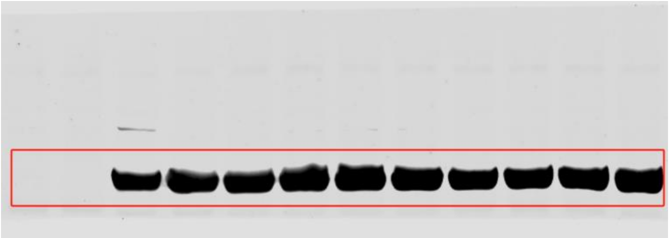

Input-GAPDH

kDa
-100
-36
-0

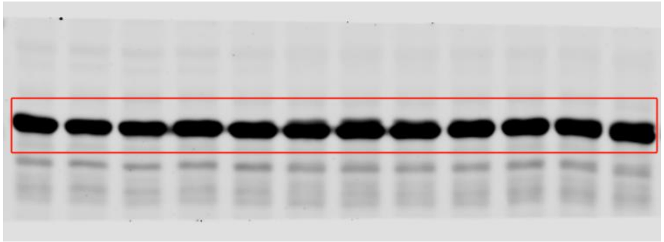

Figure 4E

	Myc-SLC3A2					
Flag-Skp2	+	-	+	-	+	-
HA-Ub	K48	K48	K63	K63	WT	WT

IP-Ub

kDa
-200
-0

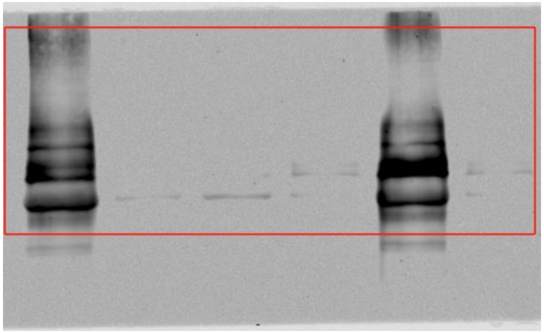

Input-Flag

kDa
-70
-48

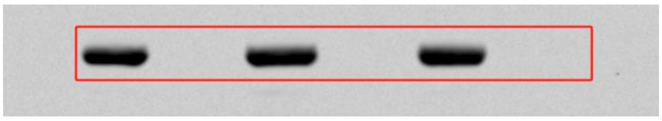

-25

Input-Myc

kDa
-110

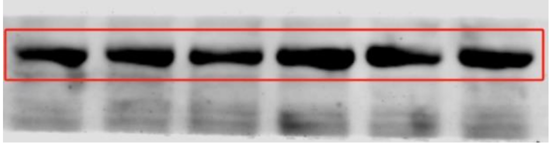

-90

-70

Input-GAPDH

kDa
-70

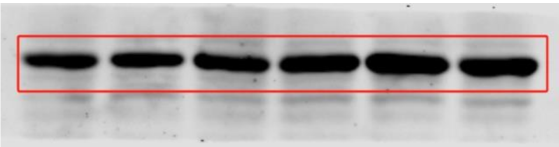

-36

-0

Figure 4F

Input-Flag

kDa
-70

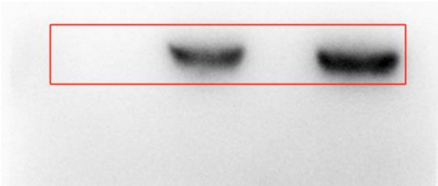

-48

-15

Flag-Skp2	-	+	-	+
MG132	-	-	-	-

Input-Myc

kDa
-100
-90

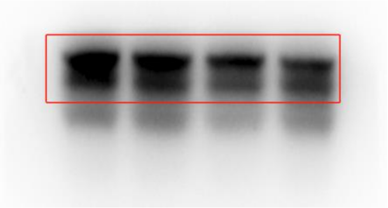

-55

Flag-Skp2	-	+	-	+
MG132	-	-	-	-

Input-GAPDH

kDa
-100

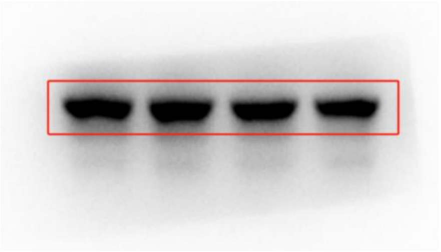

-36

-0

Flag-Skp2	-	+	-	+
MG132	-	-	-	-

IP-Ub

kDa
-200

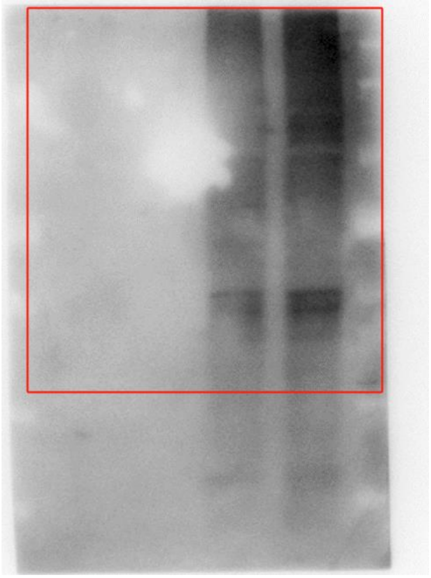

-0

Flag-Skp2	-	+	-	+
MG132	-	-	-	-

Supplemental Figure 1A

Skp2

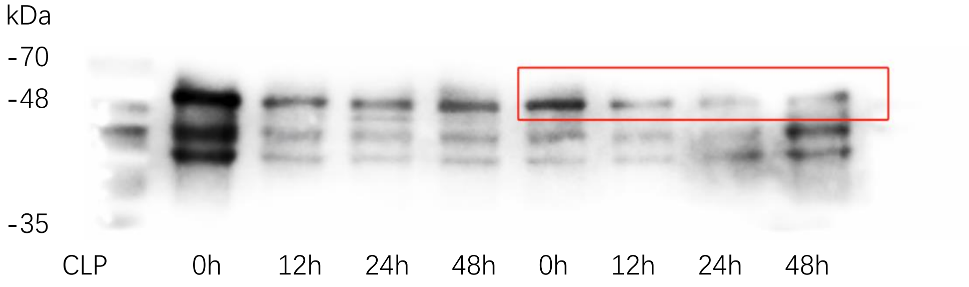

β-actin

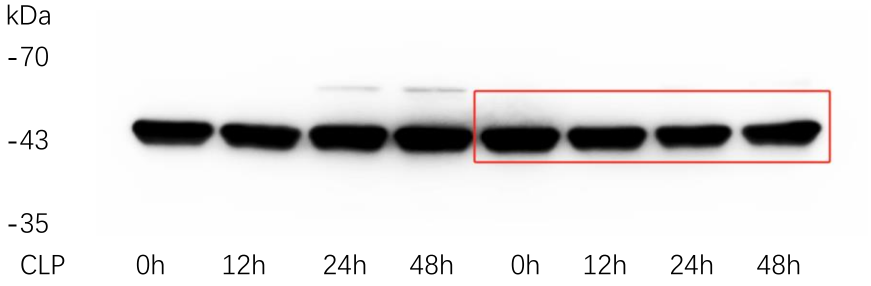

Supplemental Figure 1B

Skp2

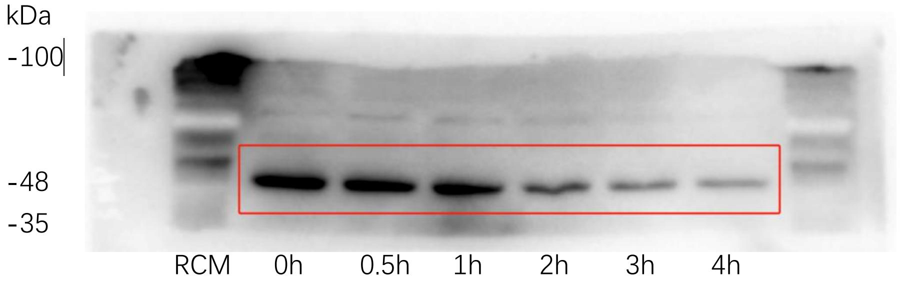

β-actin

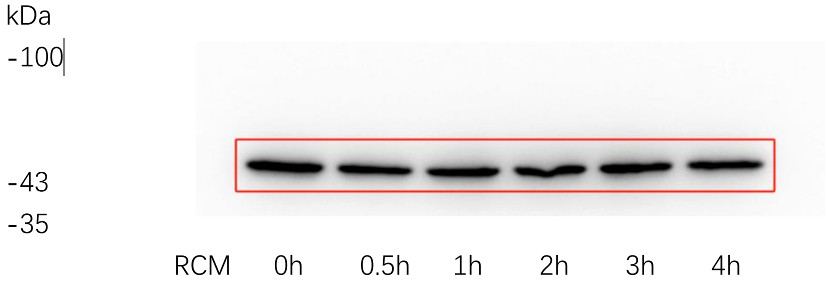

Supplemental Figure 1J

Skp2

kDa
-100
-48
-25

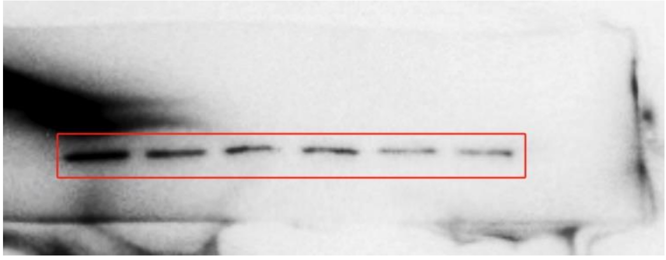

Con. LPS RCM RCM+LPS TNF-α IFN-γ

β-actin

kDa
-100
-43
-35

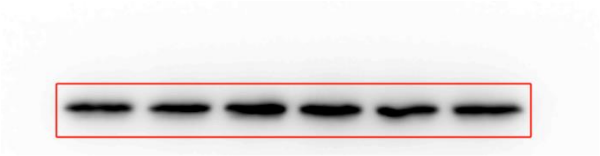

Con. LPS RCM RCM+LPS TNF-α IFN-γ

Supplemental Figure 2A

Skp2

kDa
-70
-48
-35

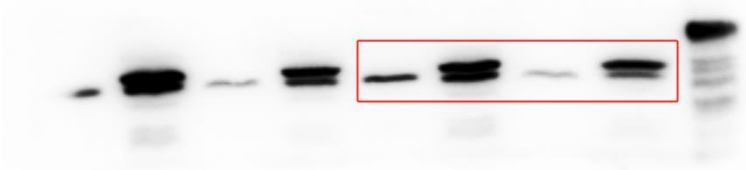

Puro Lentivirus	+	-	+	-	+	-	+	-
Skp2-3*Flag-Puro	-	+	-	+	-	+	-	+
RCM	-	-	+	+	-	-	+	+

GPX4

kDa
-35
-20
-0

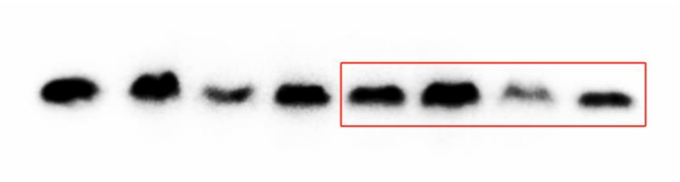

Puro Lentivirus	+	-	+	-	+	-	+	-
Skp2-3*Flag-Puro	-	+	-	+	-	+	-	+
RCM	-	-	+	+	-	-	+	+

β-actin

kDa

-70

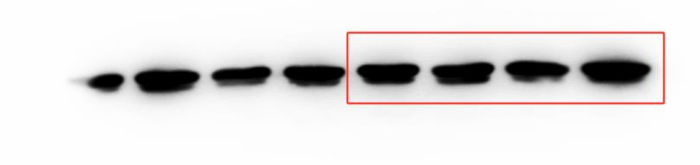

-43

-35

Puro Lentivirus	+	-	+	-	+	-	+	-
Skp2-3*Flag-Puro	-	+	-	+	-	+	-	+
RCM	-	-	+	+	-	-	+	+

Supplemental Figure 2B

Skp2

kDa

-70

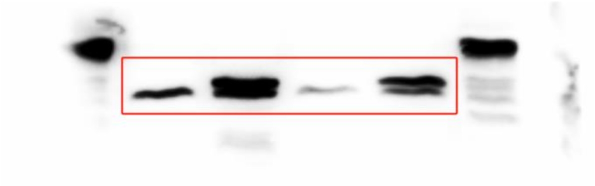

-48

-35

Puro Lentivirus	+	-	+	-
Skp2-3*Flag-Puro	-	+	-	+
Erastin	-	-	+	+

GPX4

kDa

-35

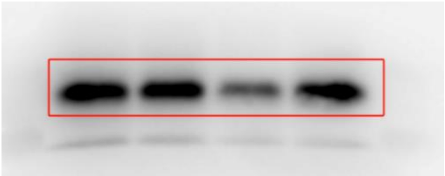

-20

-0

Puro Lentivirus	+	-	+	-
Skp2-3*Flag-Puro	-	+	-	+
Erastin	-	-	+	+

β-actin

kDa
-70
-43
-35

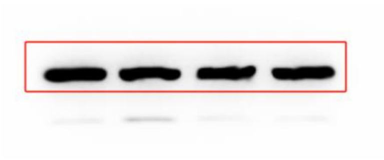

Puro Lentivirus	+	-	+	-
Skp2-3*Flag-Puro	-	+	-	+
Erastin	-	-	+	+

Supplemental Figure 2C

Skp2

kDa
-70
-48
-35

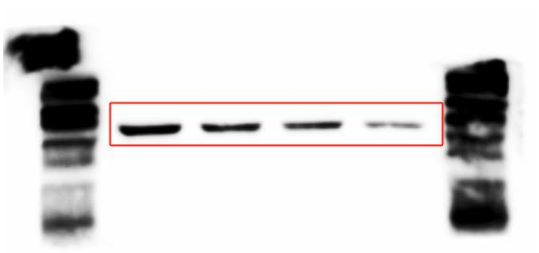

Scrambled siRNA	+	-	+	-
Skp2 siRNA	-	+	-	+
RCM	-	-	+	+

GPX4

kDa
-35
-20
-0

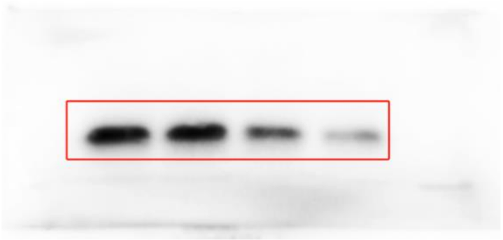

Scrambled siRNA	+	-	+	-
Skp2 siRNA	-	+	-	+
RCM	-	-	+	+

β-actin

kDa
-70
-43
-35

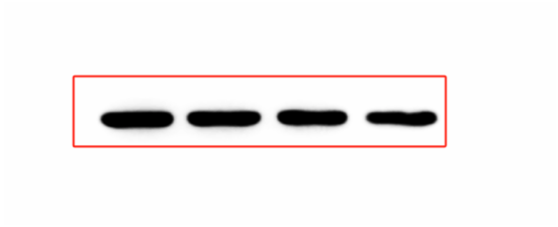

Scrambled siRNA	+	-	+	-
Skp2 siRNA	-	+	-	+
RCM	-	-	+	+

Supplemental Figure 2D

Skp2

kDa
-70
-48
-35

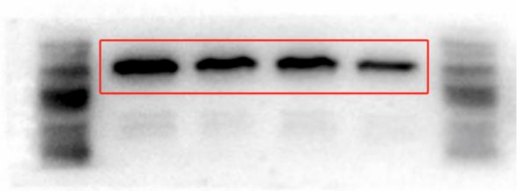

SIMP004	-	+	-	+
RCM	-	-	+	+

GPX4

kDa
-35
-20
-0

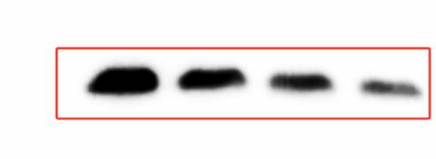

SIMP004	-	+	-	+
RCM	-	-	+	+

β-actin

kDa
-70
-43
-35

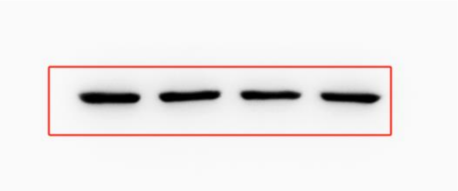

SIMP004 - + - +
RCM - - + +

Supplemental Figure 3A

GSDMD/GSDMD-N (12.5%gel)

kDa
-100
-55
-35
-0

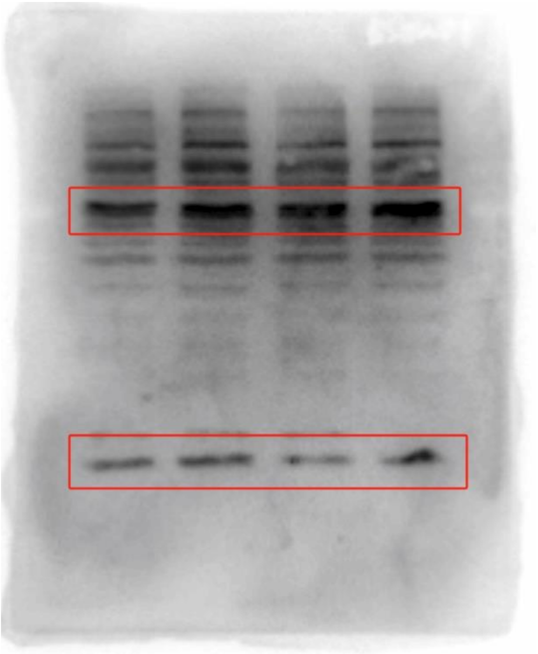

Con. LPS Erastin LPS+CTB

Skp2

kDa
-70
-48
-35

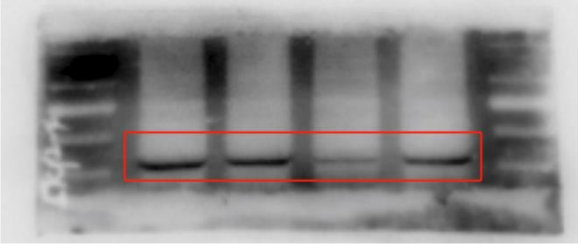

Con. LPS Erastin LPS+CTB

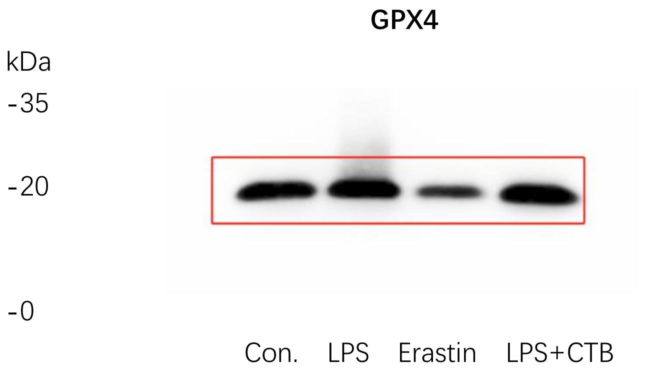

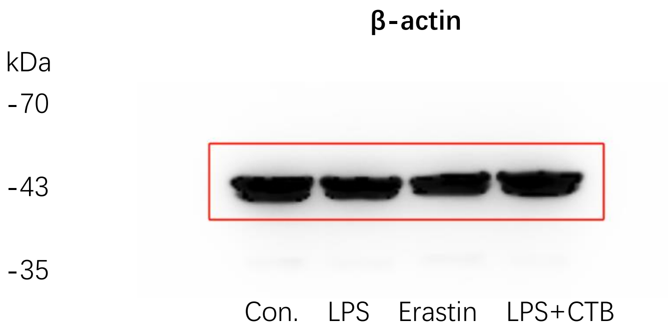

Supplemental Figure 3C

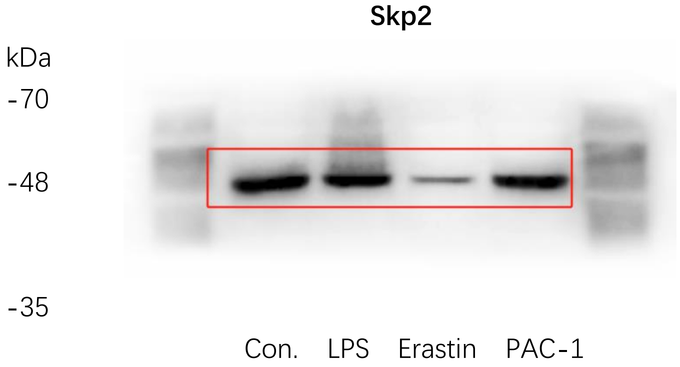

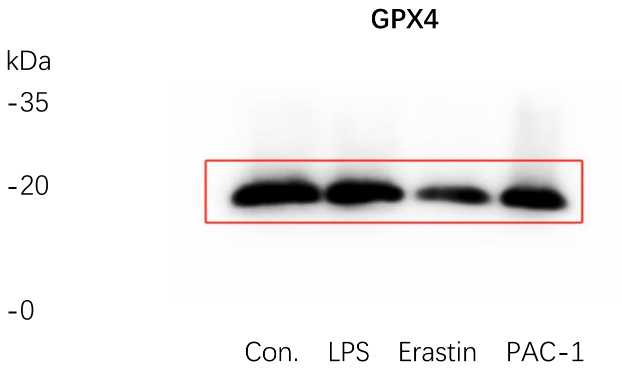

β-actin

kDa
-70
-43
-35

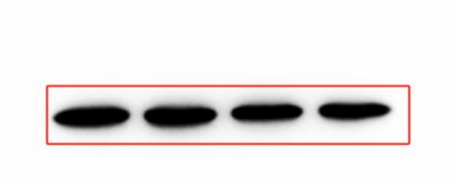

Con. LPS Erastin PAC-1

Supplemental Figure 3E

Skp2

kDa
-70
-48
-35

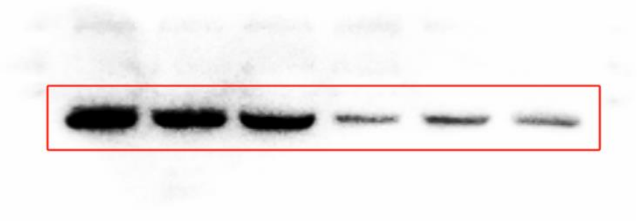

Con. Nec-1 Z-VAD Era Era+Nec-1 Era+Z-VAD

GPX4

kDa
-35
-20
-0

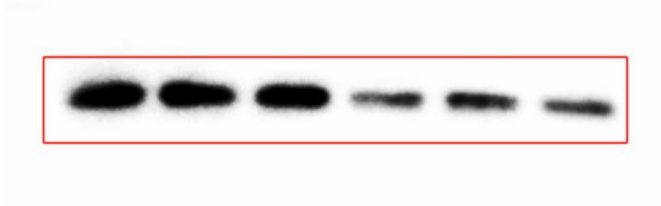

Con. Nec-1 Z-VAD Era Era+Nec-1 Era+Z-VAD

β-actin

kDa
-70
-43
-35

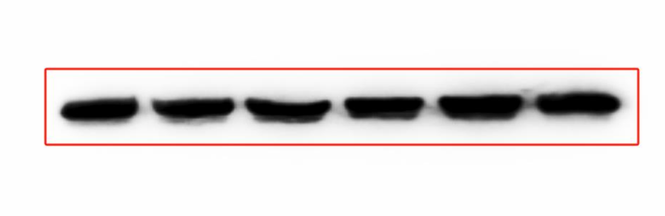

Con. Nec-1 Z-VAD Era Era+Nec-1 Era+Z-VAD

Supplemental Figure 4A

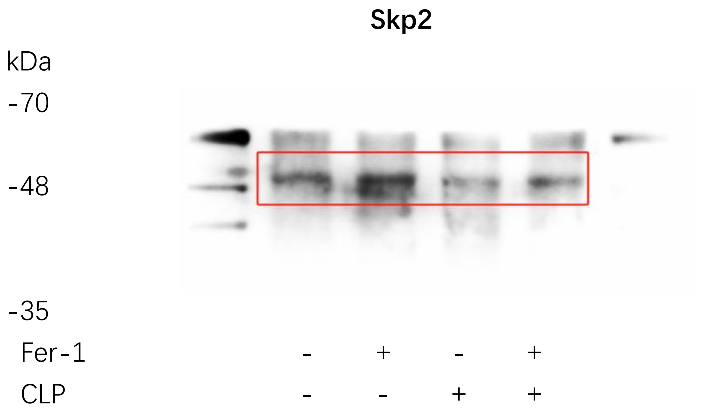

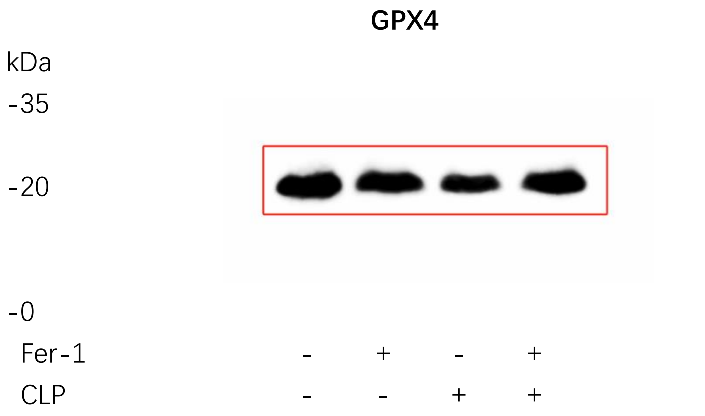

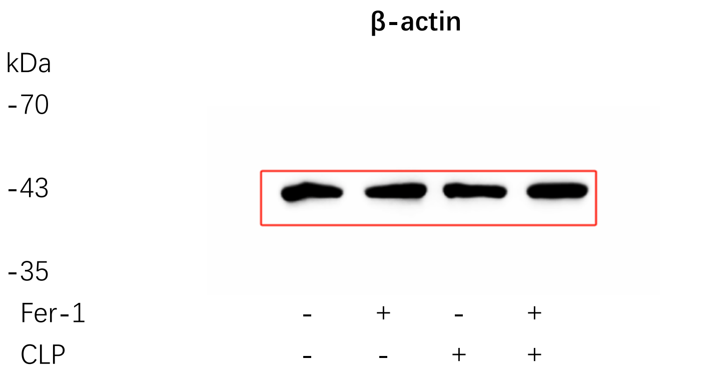

Supplemental Figure 4B

Skp2

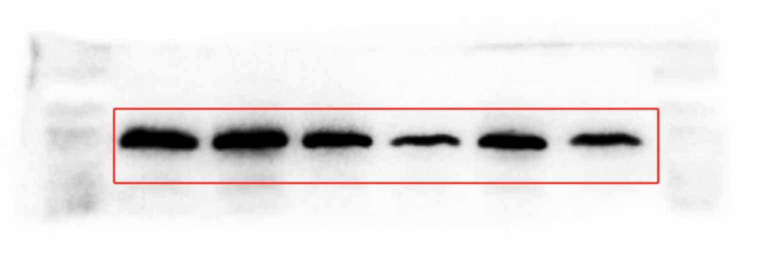

GPX4

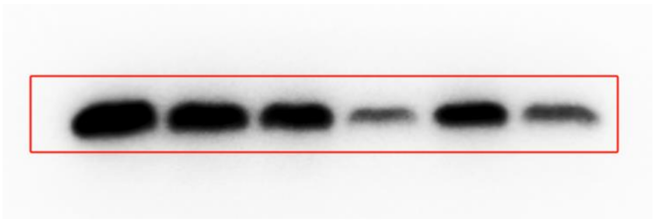

β-actin

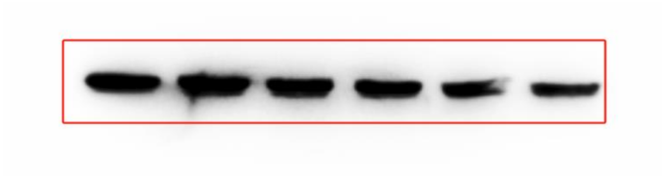

Supplemental Figure 4C

p-MEK

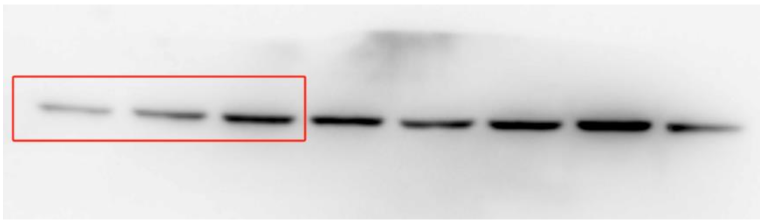

-15

Con. LPS Erastin RLS3 Con. LPS Erastin RLS3

MEK

kDa
-70
-45
-15

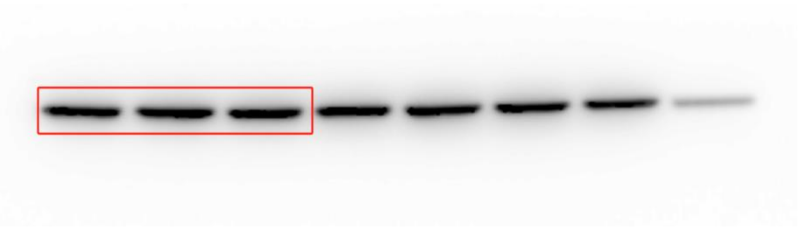

Con. LPS Erastin RLS3 Con. LPS Erastin RLS3

β-actin

kDa
-70
-43
-35

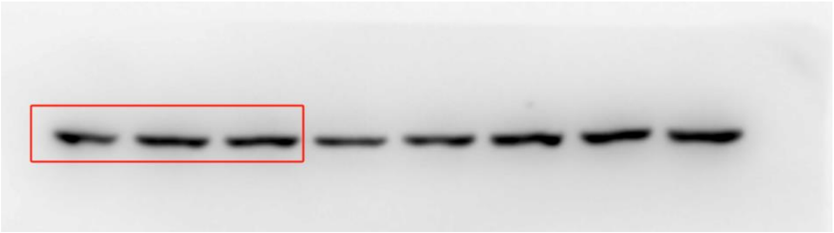

Con. LPS Erastin RLS3 Con. LPS Erastin RLS3

Supplemental Figure 5A

IP-Flag-Skp2/Anti-Flag

kDa
-200
-48
-0

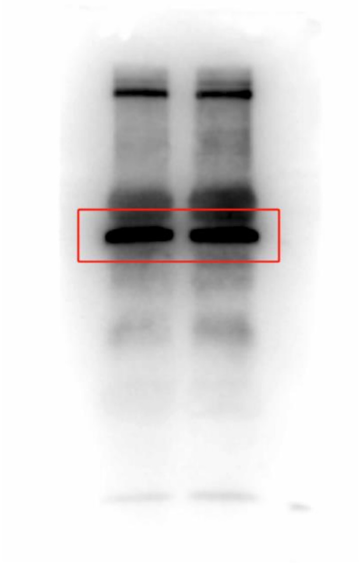

Con. RCM

IP-SLC3A2

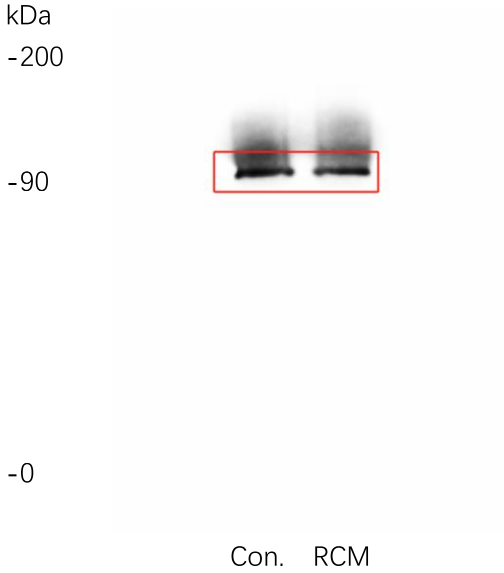

Input-Flag-Skp2

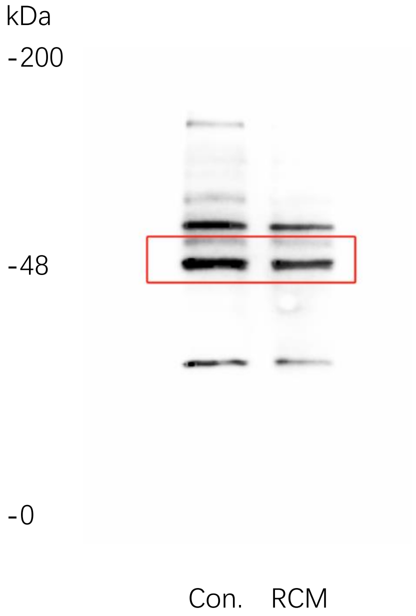

Input-SLC3A2

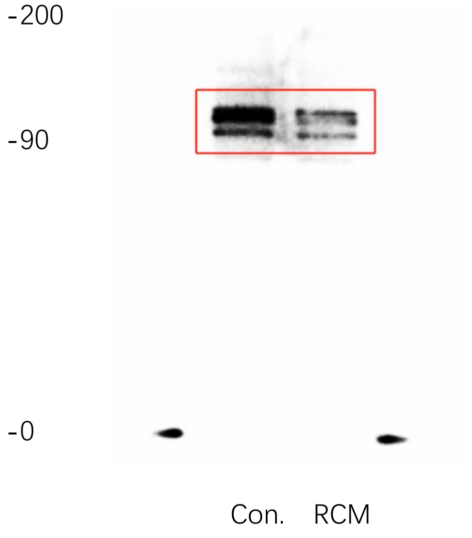

Input-β-actin

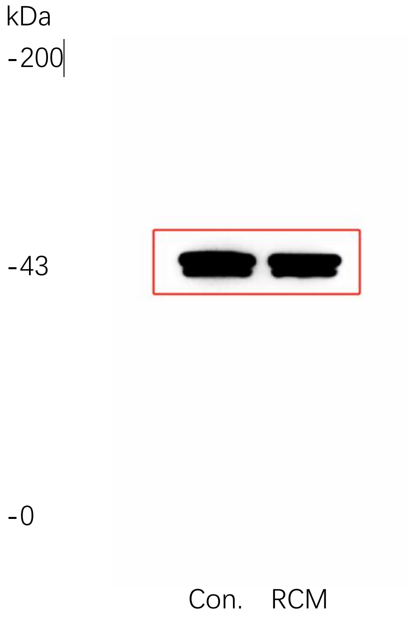

Supplemental Figure 6B

Skp2

kDa
-70
-48
-35

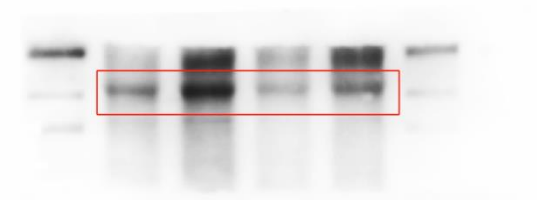

Vehicle	+	-	+	-
Skp2-LNP	-	+	-	+
CLP	-	-	+	+

GPX4

kDa
-35
-20
-0

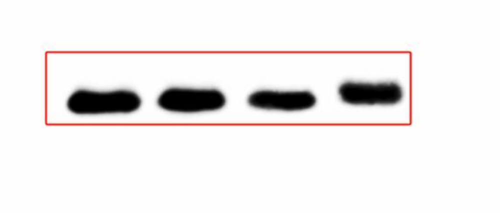

Vehicle	+	-	+	-
Skp2-LNP	-	+	-	+
CLP	-	-	+	+

β-actin

kDa
-70
-43
-35

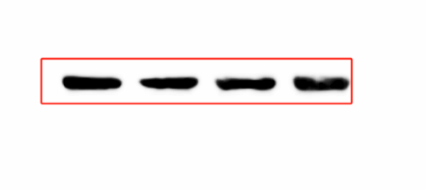

Vehicle	+	-	+	-
Skp2-LNP	-	+	-	+
CLP	-	-	+	+
